# Indoor Airborne Microbiome and Endotoxin: Meteorological Events and Occupant Characteristics Are Important Determinants

**DOI:** 10.1021/acs.est.3c01616

**Published:** 2023-07-31

**Authors:** Hesham Amin, Tina Šantl-Temkiv, Christine Cramer, Kai Finster, Francisco Gomez Real, Thorarinn Gislason, Mathias Holm, Christer Janson, Nils Oskar Jögi, Rain Jogi, Andrei Malinovschi, Ian P. G. Marshall, Lars Modig, Dan Norbäck, Rajesh Shigdel, Torben Sigsgaard, Cecilie Svanes, Hulda Thorarinsdottir, Inge M. Wouters, Vivi Schlünssen, Randi J. Bertelsen

**Affiliations:** † Department of Clinical Science, University of Bergen, 5021 Bergen, Norway; ‡ Section for Microbiology, Department of Biology, Aarhus University, 8000 Aarhus, Denmark; § Department of Public Health, Environment, Work and Health, Danish Ramazzini Center, Aarhus University, 8000 Aarhus, Denmark; ∥ Department of Occupational Medicine, Danish Ramazzini Center, Aarhus University Hospital, 8200 Aarhus, Denmark; ⊥ Faculty of Medicine, University of Iceland, 102 Reykjavík, Iceland; # Department of Occupational and Environmental Medicine, University of Gothenburg, 405 30 Gothenburg, Sweden; ∇ Department of Medical Sciences: Respiratory, Allergy, Sleep Research, Uppsala University, 751 85 Uppsala, Sweden; ○ Department of Medical Sciences: Clinical Physiology, Uppsala University, 751 85 Uppsala, Sweden; ◆ Tartu University Hospital, Lung Clinic, 50406 Tartu, Estonia; ¶ Division of Occupational and Environmental Medicine, Department of Public Health and Clinical Medicine, Umeå University, 901 87 Umeå, Sweden; †† Department of Medical Sciences, Occupational and Environmental Medicine, Uppsala University, 751 85 Uppsala, Sweden; ‡‡ Department of Occupational Medicine, Haukeland University Hospital, 5053 Bergen, Norway; §§ Centre for International Health, University of Bergen Department of Global Public Health and Primary Care, 5009 Bergen, Norway; ∥∥ Department of Anesthesia and Intensive Care, Landspitali University Hospital, 101 Reykjavik, Iceland; ⊥⊥ Institute for Risk Assessment Sciences, Faculty of Veterinary Medicine, Utrecht University, 3584 CS Utrecht, The Netherlands

**Keywords:** Northern Europe, airborne microbiome, meteorological data, 16S rRNA and occupants’ age

## Abstract

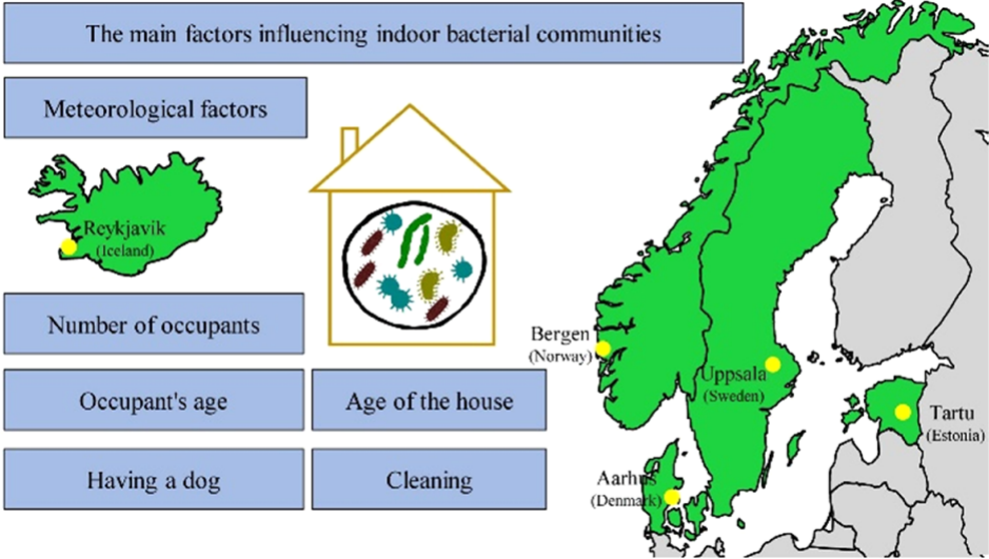

Airborne bacteria and endotoxin may affect asthma and allergies. However, there is limited understanding of the environmental determinants that influence them. This study investigated the airborne microbiomes in the homes of 1038 participants from five cities in Northern Europe: Aarhus, Bergen, Reykjavik, Tartu, and Uppsala. Airborne dust particles were sampled with electrostatic dust fall collectors (EDCs) from the participants’ bedrooms. The dust washed from the EDCs’ clothes was used to extract DNA and endotoxin. The DNA extracts were used for quantitative polymerase chain (qPCR) measurement and 16S rRNA gene sequencing, while endotoxin was measured using the kinetic chromogenic limulus amoebocyte lysate (LAL) assay. The results showed that households in Tartu and Aarhus had a higher bacterial load and diversity than those in Bergen and Reykjavik, possibly due to elevated concentrations of outdoor bacterial taxa associated with low precipitation and high wind speeds. Bergen-Tartu had the highest difference (ANOSIM *R* = 0.203) in β diversity. Multivariate regression models showed that α diversity indices and bacterial and endotoxin loads were positively associated with the occupants’ age, number of occupants, cleaning frequency, presence of dogs, and age of the house. Further studies are needed to understand how meteorological factors influence the indoor bacterial community in light of climate change.

## Abstract 2

Minimal research exists on the factors influencing the indoor bacterial community. Despite their proposed importance for health, here we report environmental factors influencing the composition of the indoor bacterial communities.

## 1 Introduction

Today most humans have largely removed themselves from the outdoor environments in which they evolved, and spend >90% of their time indoors, i.e., in houses, offices, and schools.^1^ Exposure to bacterial communities inside the indoor environment can impact human health.^2^ Early life exposure to increased microbial load and diversity has been shown to be protective against allergic outcomes such as allergic asthma.^3^ Researchers have used endotoxin concentrations as a proxy measure of bacteria exposure to understand the link between bacterial exposure and health outcomes in farming and nonfarming populations.^4,5^ Using next-generation sequencing techniques, it has been shown that specific bacterial taxa are associated with asthma and atopy in both children and adults.^6−8^ Studies of differences in house dust microbiome composition between farm and nonfarm homes of Finnish and German birth cohorts showed that the protective microbiome against asthma and atopy had a low abundance of *Streptococcaceae* relative to outdoor-associated bacterial taxa such as *Sphingobacteria* and endotoxin-producing bacteria belonging to the *Alphaproteobacteria* class.^9^

The indoor air environment is populated by different bacterial communities that originated from different sources, including human and animal occupants as well as outdoor air.^10−12^ Despite the proposed importance of the indoor microbiome on health, the relative contributions of these sources, as well as factors influencing the composition of the indoor airborne bacterial communities, are largely unknown. Understanding the determinants of the indoor bacterial community is pivotal to be able to influence the indoor microbiome and ultimately prevent negative health effects. A better understanding of the relationship between exposure to specific microorganisms and asthma and allergies is urgently needed, particularly in regions such as northern Europe, where the prevalence of allergic diseases and asthma has increased dramatically in recent decades.^13^

Hitherto, studies of the indoor microbial community and environmental determinants associated with the indoor environment have been limited to single geographical sites and small sample sizes (∼100).^14−18^ Many environmental determinants such as building materials, occupant behaviors, and climate factors such as the precipitation rate and relative humidity affecting outdoor bacterial taxa are rather uniform within single geographical sites.^19^ To identify the factors that affect indoor bacterial community variation, studies on a regional scale with complementary and comprehensive environmental data are required. Therefore, we studied the indoor bacterial air community in more than 1038 homes in ECRHS III. The goals of our study, in which we focused on the bacterial community and endotoxin, were (1) to make an inventory of the indoor airborne bacterial community composition, including α and β diversity, in five medium-sized northern European cities, and (2) to identify environmental factors associated with the composition of the bacterial communities and endotoxin concentration indoor.

## 2 Materials and Methods

### 2.1 Study Populations

The present study initially comprised 1080 homes of the participants of the ECRHS III from Aarhus (Denmark), Bergen (Norway), Reykjavik (Iceland), Tartu (Estonia), and Uppsala (Sweden). The ECRHS (European Community Respiratory Health Survey) is an international multicentre population-based study aiming to determine the prevalence of and risk factors for the development of asthma and allergic diseases in adults living in Europe and Australia.^20^ The participants were between 22 and 44 years at baseline around 1990. From 2011 to 2014, all of the participants invited for ECRHS III clinical examinations and interview questionnaires were asked to collect settled dust using an electrostatic dust fall collector (EDC) (Supporting Figure 1). Except for the Tartu study center, all participants filled in a short questionnaire related to the EDC (EDC questionnaires). The samples where participants reported that the EDC fell on the floor (23 samples) as well as samples that did not reach defined quality standards (better number of reads) in 16S rRNA amplicon sequencing (19 samples) were removed from the analysis. As a result, the total number of persons and samples included in the analysis was 1038. Information about environmental determinants was extracted from ECRHS III interviews and the EDC questionnaires. Local ethics committees at each center approved the study protocols. For detailed information about questionnaires, we refer to the official ECRHS website: http://www.ecrhs.org/. The study centers, number of participants from each center, and other environmental determinants of the study object are listed in Table 1.

**Table 1:** Characteristics of the Study Population

	Aarhus (*N* = 160)	Bergen (*N* = 300)	Reykjavik (*N* = 346)	Tartu (*N* = 84)	Uppsala (*N* = 148)	Total (*N* = 1038)^a^
season of sampling						
summer	21	123	150	70	63	427 (41.5%)
winter	139	175	194	14	78	600 (58.5%)
no. of occupants in the home						
one	23	34	33	15	17	122 (12.6%)
two or more	137	216	312	69	106	840 (87.4%)
occupant’s age mean (SD)	53 (±6.5)	53 (±6.8)	55 (±7.1)	52 (±7.1)	56 (±7.2)	54 (±7.0)
dog in bedroom	31	25	47	6	10	119 (12.4%)
cat in bedroom	15	23	36	19	18	111 (11.4%)
kitchen fan use						
never	8	7	89	36	4	144 (15.2%)
sometimes	37	66	164	20	36	323 (34.1%)
all of the time	109	174	89	28	80	480 (50.7%)
window open at night						
never	94	97	56	63	83	393 (42.3%)
sometimes	12	42	102	12	21	189 (20.4%)
all of the time	53	107	187	9	18	374 (40.2%)
cleaning frequency						
less than 1 time per week	60	62	103	13	27	265 (27.7%)
1–3 times per week	83	163	188	63	86	583 (60.8%)
4–7 times per week	17	21	54	8	10	110 (11.5%)
use of bleach	26	106	86	1	22	241 (33.4%)
use of ammonia	23	85	6	1	9	124 (14.7%)
house age (years, mean (SD))	50 (±36)	41 (±34)	34 (±22)	41 (±26)	49 (±28)	41 (±30)
mattress age (years, mean (SD))	7.1 (±5.5)	7.7 (±5.8)	8.0 (±5.5)	7.7 (±8.8)	6.4 (±5.1)	7.5 (±5.9)
central heating	143	11	338	51	102	645 (67.1%)
ducted heating	5	23	1	1	24	55 (5.7%)
electric heating	11	238	0	40	31	320 (33.3%)
open coal heating	11	78	0	7	16	112 (11.6%)
radiator in bedroom	138	5	326	53	117	639 (66.5%)
air condition	0	41	5	14	10	70 (7.3%)
airbrick bedroom	34	5	0	1	78	118 (11.3%)
damp spots in bedroom	8	7	11	0	1	27 (2.9%)
condensation on window	76	48	49	30	22	225 (23.5%)
mold odor	18	10	23	11	4	66 (6.9%)
mold	43	35	34	23	16	151 (15.8%)
water damage	45	65	98	43	39	290 (31%)
no. of rooms						
one	1	2	0	8	1	12 (1.3%)
two	8	12	10	18	6	54 (5.6%)
three or more	151	235	334	58	115	894 (93.1%)
floor level						
ground floor	1	20	9	0	2	32 (3.3%)
first floor	86	94	147	26	47	400 (41.6%)
higher than first floor	73	136	189	58	73	529 (55.1%)
rug in bedroom	33	52	49	53	69	256 (26.7%)
fitted carpet in bedroom	40	13	3	9	3	68 (7.1%)
bedroom size (m^2^, mean (SD))^b^	15 (±7.1)	13 (±3.9)	15 (±5.8)		15 (±4.9)	14 (±5.4)
floor heating^b^	22	34	18		1	75 (9.4%)
bedroom wallpaper^b^	4	88	10		102	204 (27.4%)
painted fiberglass^b^	15	72	4		9	120 (16.4%)
wall vent^b^	34	156	233		31	454 (52%)
ceiling exhaust^b^	2	6	14		17	39 (4.8%)
house type^b^						
apartment building	27	82	123		44	276 (30.8%)
detached house	76	138	116		65	395 (44.1%)
farmhouse	8	5	5		4	22 (2.4%)
terraced house	38	63	83		19	203 (22.7%)
precipitation rate (mm/day, mean (SD))	1.8 (±0.94)	8.3 (±3.7)	4.4 (±2.3)	1.9 (±0.70)	2.2 (±1.1)	4.7 (±3.6)
temperature (C°, mean (SD))	6.6 (±4.8)	2.5 (±5.2)	4.1 (±3.6)	6.0 (±7.1)	3.7 (±7.9)	4.1 (±5.5)
relative humidity (%, mean(SD))	91 (±3.6)	90 (±4.9)	86 (±5.8)	89 (±6.8)	90 (±8.1)	89 (±6.1)
wind speed (m/s, mean (SD))	6.2 (±0.75)	3.1 (±0.53)	5.3 (±1.5)	5.2 (±0.84)	2.2 (±0.21)	4.3 (±1.7)

a Information was missing for season (*n* = 11, 1%), All of the other characteristics were missing for around 80 participants (7–9%).

b Variables extracted from the EDC questionnaire.

### 2.2 Dust Sampling

Between March 2011 and January 2014, settled airborne dust was collected in participants’ bedrooms over a 14-day period using EDCs (Supporting Figure 1) with an exposure area of 209 cm^2^. The EDCs were placed 1.5 m above the floor.^21^ The participants were instructed to return the EDCs by mail, along with the EDC questionnaires. All EDC samples were stored at −20 °C until dust extraction.

### 2.3 Dust, Endotoxin, and DNA Extraction

In 2022, the EDC clothes were handled as described previously, where dust, endotoxin, and DNA extraction from EDC clothes were optimized to obtain a comprehensive representation of the airborne bacterial communities.^22^ For a detailed description of the dust, endotoxin, and DNA extraction, see the methods section in the Supporting Information.

### 2.4 16S rRNA Amplicon Sequencing

16S rRNA genes from the samples (including 35 control samples and 20 PCR controls) were amplified using the bacteria-specific primers targeting the V3 and V4 regions of the 16S rRNA gene. The Illumina protocol (16S Metagenomic Sequencing Library Preparation) was used for amplification of the 16S rRNA gene. The detailed description of the primers and the protocol for 16S rRNA gene sequencing is described in the methods section in the Supporting Information.

### 2.5 Quantitative PCR

The qPCR reactions targeting 16S rRNA genes were carried out using an MX3005p qPCR machine (Agilent, Santa Clara, CA). For a detailed description of the primers, the qPCR reaction components, and thermal cycling conditions, see the methods section in the Supporting Information.

### 2.6 LAL Assay

Each extract was diluted 50 times in PFW before analysis with the quantitative kinetic chromogenic LAL assay to overcome the masking effect of Tween 20 on the assay^22^ (Kinetic-QCL 50–650 U kit, Lonza, Walkersville, Maryland). Endotoxin from *Escherichia coli* O55:B5 was used as a standard. To create a standard curve, 13 serial dilutions were employed, covering a range of values between 25 and 0.006 EU/mL. The cut-off signals (*V*_max_) of the kinetic LAL Assay were defined as the average of the assay blanks plus two times the standard deviation of these blanks. The results were presented in EU m-2 units.

### 2.7 Bioinformatic and Statistical Analysis

All sequence data processing and statistical analyses were carried out in R version 4.2.1.^23^ The raw data processing is described in detail in the Supporting Information.

Microbiome version 1.15.0^24^ was used to assess α bacterial diversity (Shannon index, which reflects both richness and the relative abundance of each taxon), and bacterial richness (observed number of ASVs). The relative abundances of Gram-positive and Gram-negative bacteria were assigned, based on relative abundances of phyla across samples from the five cities (Supporting Table 1).To identify specific bacterial taxa (genus level) whose abundances significantly differ between different environmental determinants (e.g., city and season), we applied analysis of compositions of microbiomes with bias correction (ANCOM BC) version 1.6.2.^25^ We removed genera that accounted for less than 0.01% relative abundance and adjusted for the variables that showed association with Analysis of similarity (ANOSIM) test. The remaining taxa are affiliated to 201 bacterial genera. The ANOSIM test from the vegan package version 2.5-7^26^ that is based on the Aitchison dissimilarity matrix was used to compare bacterial community structures between different environmental determinants. Continuous variables such as age of the house and the occupants’ age were dichotomized based on the median.

The sampling period was split into two seasons, based on the monthly average temperature in the five cities obtained from the weather-base website (https://www.weatherbase.com/). The coldest months were assigned to winter (November, December, January, February, March, April), and the warmest months were assigned to summer (May, June, July, August, September, October).

To study the association between normally distributed dependent variables, i.e., bacterial diversity (Shannon index, Supporting Figure 2A) and bacterial richness (Number of bacterial taxa, Supporting Figure 2B), and independent variables, i.e., environmental determinants, we used multiple linear regression (stats package version 4.0.4^23^) based on two approaches to ensure robust regression analysis. In the first approach, we performed univariate analysis for all independent variables, and in the next step, we ran a multivariate model including the variables that showed associations (*P* ≤ 0.25 as arbitrary value) with the dependent variables.

In the second approach, we included the environmental determinants in three consecutive models. For each model, variables that showed association (*P* ≤ 0.25) with the dependent variables were kept in the model. In the first model, we included key determinants (city and season), while in the second model, we further included occupant and occupant-related behavior determinants (the presence of dog and cat, the number of occupants, the occupant’s age, and cleaning frequency). The third model considers indoor factors such as house age, type of heating system, presence of mold, condensation on the window, and ventilation. The reason behind the sequence of the models mentioned is that we expected that key determinants would be the ones with the strongest effect on indoor bacterial profiles, followed by occupant and indoor determinants based on the literature.^1,27^

To study the association between non-normal distributed dependent variables (bacterial load (16S rRNA gene copies/m^2^, Supporting Figure 2C) and endotoxin load (EU/m^2^, Supporting Figure 2D)) and environmental determinants, we used quantile regression from package “quantreg” version 5.86^28^ and followed the same two approaches as for the multivariate linear regression models mentioned earlier.

### 2.8 Meteorological Data

The monthly average meteorological data for the precipitation rate (mm/day), temperature (C°), relative humidity (%), and wind speed (m/s) for each sample were extracted from the NASA Langley Research Center POWER Project (https://power.larc.nasa.gov/) based on the city of sample collection and the EDC opening date reported by the study participants between 2011 and 2014. The precipitation rate (mm/day) represents the total depth of rainwater (mm) for 24 h.

## 3 Results

### 3.1 Quality Filtering and Study Characteristics

After quality filtering and downsampling to 20,000 reads per sample, a total of 1038 EDCs from Aarhus (*n* = 160), Bergen (*n* = 300), Reykjavik (*n* = 346), Tartu (*n* = 84), and Uppsala (*n* = 148) were included in the analysis. When performing analyses utilizing the EDC questionnaire, the Tartu samples were excluded since this questionnaire was not filled out by the Tartu participants. Thus, yielding a subgroup of 954. The characteristics of the study population based on the ECRHS III interview, the EDC questionnaire, and meteorological data during sampling the indoor dust are presented in Table 1.

### 3.2 Bacterial Load

In the first approach based on the univariate regression (Supporting Table 2), the multivariate quantile regression model showed the following determinants to be significant: cities, number of occupants, and occupant age (Supporting Table 3). Similar results were shown when the determinants were introduced in three consecutive models. Additionally, we found that cleaning more than 4 times per week was associated with a higher bacterial load compared to cleaning less than 1 time/week (Supporting Table 4).

**Figure 1:**
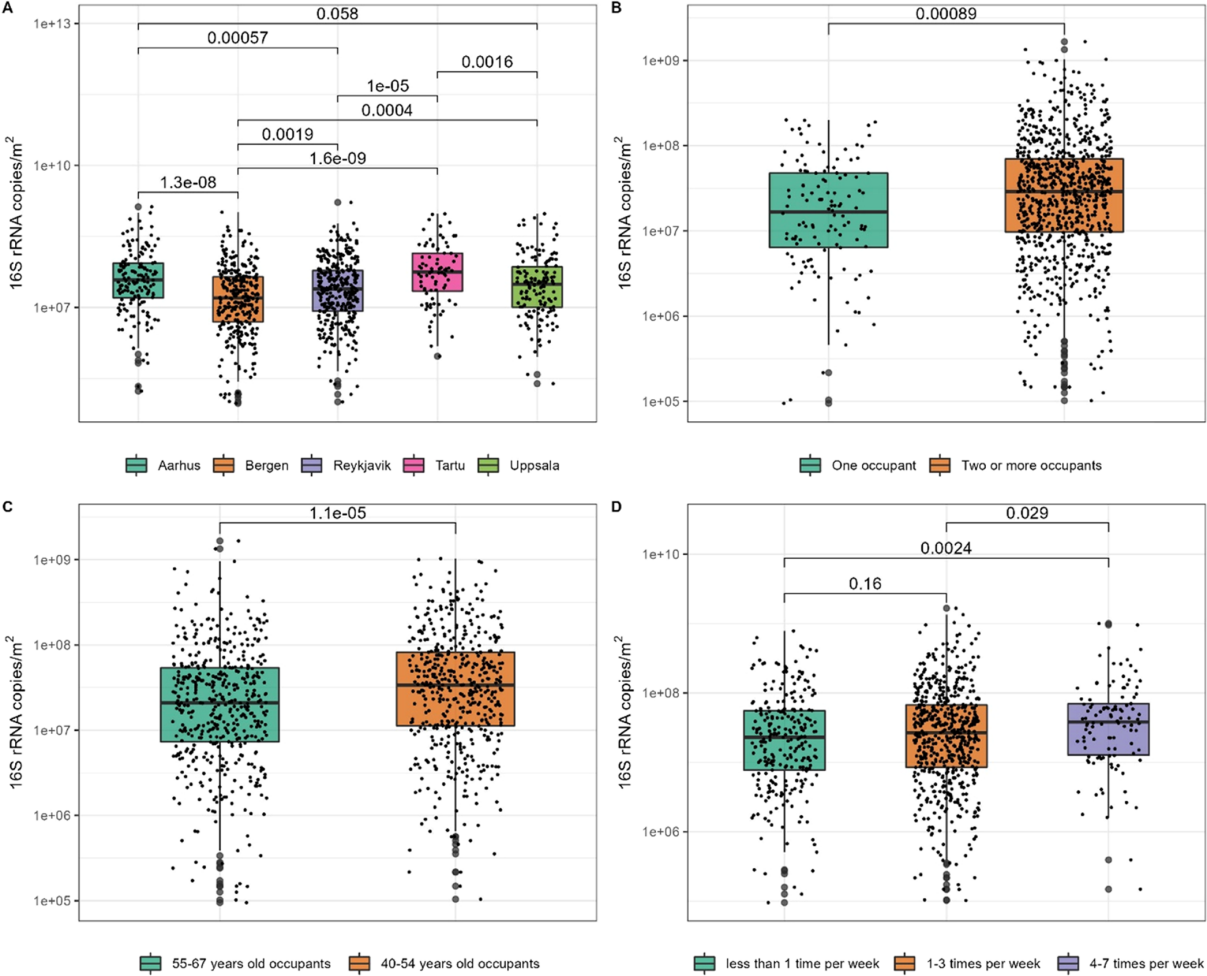
Boxplot of qPCR results of (A) cities’ households, (B) number of occupants per household (one vs two or more), (C) older occupant age group (55–67 years old) compared vs younger age group (40–54 years old), (D) cleaning frequency. *P* values based on pairwise sample comparison in Wilcoxon signed-rank test. Only the significant pairwise comparison is shown.

Bergen households showed significantly lower bacterial load compared to those from other Nordic cities. There was no significant difference in bacterial load between Tartu and Aarhus and between Reykjavik and Uppsala households (Figure 1A). Reporting more than 1 person in the house was significantly associated with higher bacterial load (*P* = 0.01) (Figure 1B). The occupants within the youngest age group (40–54 years old) showed a significantly higher bacterial load compared to the older age group (55–67 years old) (Figure 1C). A high cleaning frequency was associated with a significantly higher bacterial load (Figure 1D).

### 3.3 Bacterial Diversity and Richness

With the first approach to study the association between indoor determinants and bacterial diversity (Shannon index) and richness (observed number of ASVs), based on the univariate regression (Supporting Table 5), the multivariate regression revealed that study site (the cities), keeping a dog in the bedroom, number of occupants, occupants’ age, and age of the house to be significantly associated with both indices (Supporting Table 6). Season, condensation of water on window, and cleaning frequency (less than one time per week vs 4–7 times per week) showed significant association with the Shannon index only (Supporting Table 6).

Similar results were shown when the determinants were introduced in three consecutive models. We further found that the presence of mold was associated with increased bacterial diversity and that a rug in the bedroom increased the number of bacterial taxa (Supporting Table 7).

In a complete case analysis with data from both ECRHS III main interviews and EDC questionnaires, we found that bedroom size was significantly associated with increasing bacterial richness and diversity, while wall vent was associated with a decrease in bacterial diversity (Supporting Table 8).

**Figure 2:**
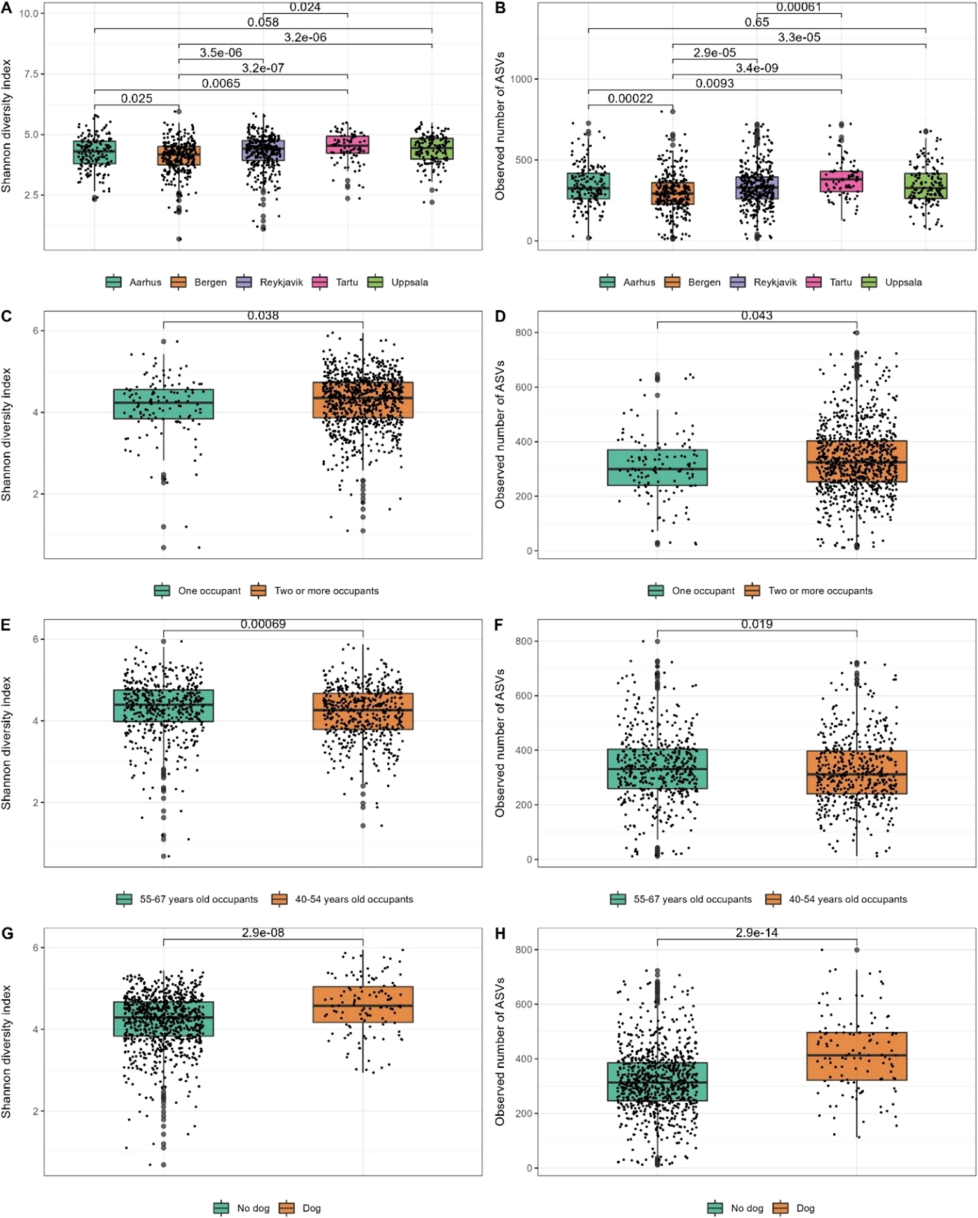
Box plots of α bacterial diversities indices. (A) Shannon index and (B) observed number of ASVs for the five cities’ households. (C) Shannon index and (D) observed number of ASVs for the number of occupants (one vs two or more). (E) Shannon index and (F) observed number of ASVs for older occupant age group (55–67 years old) vs younger age group (40–54 years old). (G) Shannon index and (H) observed number of ASVs for dog in bedroom (no vs yes). *P* values are derived from pairwise sample comparison in Wilcoxon signed-rank test (only reported for statistically significant pairwise comparison).

In terms of Shannon index and number of bacterial ASVs, Bergen households had the lowest bacterial diversity while Tartu households had the highest bacterial diversity and bacterial richness (Figure 2A,B). The number of occupants in the house (Figure 2C,D) was significantly associated with both bacterial diversity and bacterial richness. Older age of the occupants (Figure 2E,F) and the presence of a dog in the bedroom (Figure 2G,H) were both associated with increased bacterial richness and diversity.

### 3.4 Dissimilarity of Bacterial Communities (β Diversity)

Aitchison’s dissimilarity matrix, as well as the ANOSIM test for categorical variables and Mantel tests for continuous variables were used to investigate differences in the composition of the airborne bacterial community composition as a function of environmental determinants. We found a statistically significant difference in β diversity between all five cities’ households using pairwise comparisons (Supporting Table 9). The pairwise comparison between cities showed the highest difference in β diversity between Bergen and Tartu households (ANOSIM *R* = 0.304, *P* = 0.001) followed by Reykjavik vs Tartu households (ANOSIM *R* = 0.203, *P* = 0.001) while the lowest difference in β diversity was found between Bergen and Reykjavik households (ANOSIM *R* = 0.042, *P* = 0.001). The difference in the β diversity between all cities’ households was significant (*R* = 0.1803, *P* value = 0.001).

The presence of a dog in the bedroom was associated with a significant difference in β diversity (ANOSIM *R* = 0.296, *P* = 0.001), whereas the presence of a cat in the bedroom was not (ANOSIM *R* = 0.0507, *P* = 0.09). Determinants which also showed significant association with β diversity of the indoor microbiomes were cleaning frequency, having the window open during night, wall vent, having a rug in the bedroom, and the number of rooms in the house (Supporting Table 9). Mantel test for continuous variables revealed that the occupants’ age (Mantel *R* = 0.04, *P* = 0.002) and the age of the house (Mantel *R* = 0.04, *P* = 0.01) showed significant association with β diversity of indoor microbiome (Supporting Table 10).

### 3.5 Bacterial Community Composition and Differential Abundance Analysis

The indoor airborne bacterial communities in the five cities’ households were dominated by five phyla: *Firmicutes*, *Proteobacteria*, *Actinobacteria*, *Myxococcota*, and *Bacteroidetes*, which made up about 97% of the total communities (Supporting Figure 3A). We found higher relative abundance for *Actinobacteria* in Bergen and Reykjavik households, whereas the relative abundance of *Proteobacteria* was higher in Aarhus and Tartu households. Family-level composition showed that Gram-negative bacterial families such as *Rhodobacteraceae* and *Sphingomonadaceae* are more abundant in Aarhus and Tartu households (Supporting Figure 3B) than in Bergen, Reykjavik, and Uppsala households. The three most abundant bacterial families in the five cities were Gram-positive: *Micrococcaceae*, *Staphylococcaceae*, and *Corynebacteriaceae* (Supporting Figure 3B). On the genus level, the three most abundant genera were *Micrococcus*, *Staphylococcus*, and *Corynebacterium*, which belong to the three most abundant bacterial families (Supporting Figure 3C).

For the determination of the differential abundance of bacterial genera, we focused on the environmental determinants that showed significant association with β diversity (ANOSIM and Mantel tests) as well as other determinants such as water damage, season of sampling, and number of occupants, based on the literature.^1,27,29^

**Figure 3:**
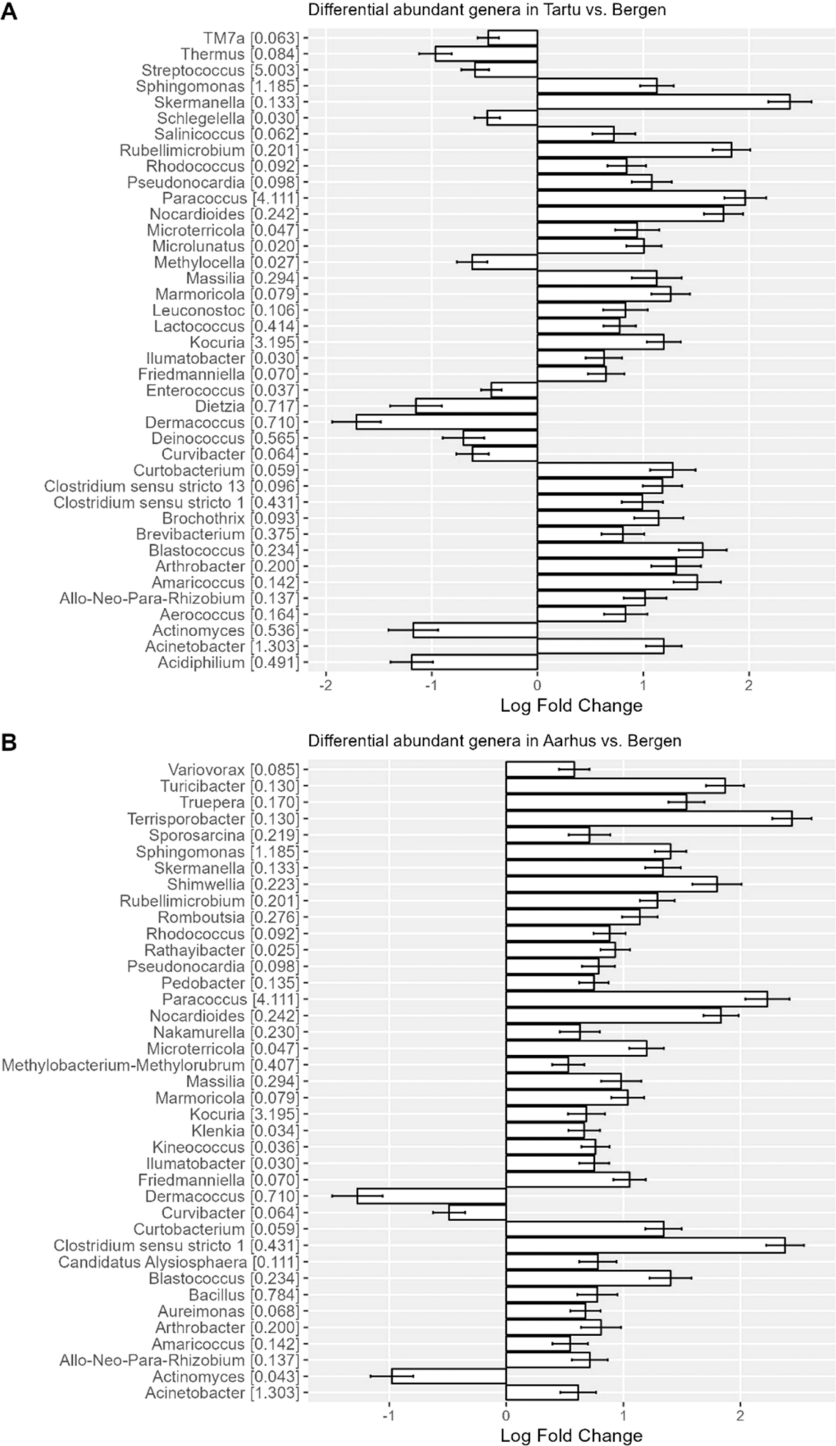
Differential abundant bacterial genera (A) in Tartu compared to Bergen households and (B) in Aarhus compared to Bergen households. Number in parentheses shows the relative abundance of the bacterial genera in the total number of samples. Positive log fold changes indicate an increase, and negative log fold changes indicate a decrease in the abundance of bacterial taxa compared to the reference group.

We found 40 out of 201 bacterial genera to be differentially abundant between Bergen and Tartu households (Figure 3A). Many of the bacterial genera which were differentially abundant between Bergen and Tartu households were also differentially abundant between Bergen and Aarhus households (Figure 3B). In general, members of the phylum *Proteobacteria*, such as *Acinetobacter, Skermanella*, *Paracoccus*, and *Sphingomonas* genera, were significantly higher in abundance in Aarhus and Tartu households compared to other cities’ households. Other pairwise differential abundance analysis between the five cities’ households can be found in Supporting Figures 4–7.

**Figure 4:**
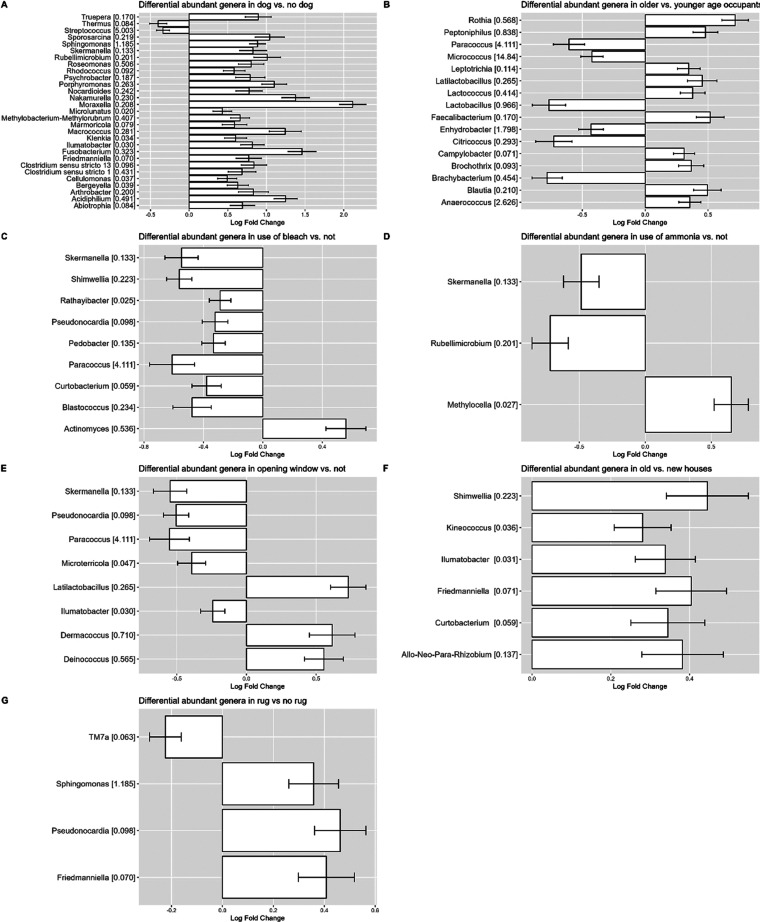
Differential abundant bacterial genera: (A) presence of dog in bedroom compared to absence, (B) older occupant age group compared to younger age group, (C) using bleach compared to not using bleach, (D) using ammonia compared to not using ammonia, (E) opening window compared to not opening the window, (F) old compared to new houses (G) presence of rug in bedroom compared to absence. Positive log fold changes indicate an increase, and negative log fold changes indicate a decrease in the abundance of bacterial taxa compared to the reference group.

The determinants related to the occupants which showed association with genera that expressed differential abundances were dog in bedroom and occupants’ age. The presence of a dog in the bedroom was associated with a higher abundance of 25 bacterial genera (Figure 4A). There was no difference in the abundance of genera when a cat was present in the bedroom. Ten bacterial genera were more abundant within the older age group (55–67 years old) and 6 genera were less abundant compared to the younger age group (40–54 years old) (Figure 4B). Occupant behavior, such as cleaning frequency, did not affect the composition of the bacterial communities. However, the use of cleaning agents such as bleach and ammonia was associated with the abundance of nine and three bacterial genera, respectively (Figure 4C,D). Opening the window at night was associated with the abundance of several bacterial genera. Having the window open all the time compared to never was associated with differences in the abundance of 8 genera (Figure 4E). The indoor determinants associated with differentially abundant genera were house age and having a rug in the bedroom. Houses that were >35 years old showed 6 more abundant genera than houses that were <35 years old (Figure 4F). The presence of a rug in the bedroom was associated with an increase in the abundance of three bacterial genera (Figure 4G).

### 3.6 Endotoxin Load

Out of 1038 samples, extracts from 758 samples (73%) had endotoxin concentrations above the background level (unexposed EDC cloths). 16 covariates were identified from the univariate analyses (Supporting Table 11). Using the first approach, only cities and age of the house showed significant association with endotoxin load (Supporting Table 12). These results were confirmed by the second approach, in which the environmental determinants were introduced in three consecutive models. (Supporting Table 13). A sensitivity analysis with complete data from both the ECRHS III main interview and the EDC questionnaires (without Tartu) showed that a dog in the bedroom was significantly associated with a higher endotoxin load (Supporting Table 14).

**Figure 5:**
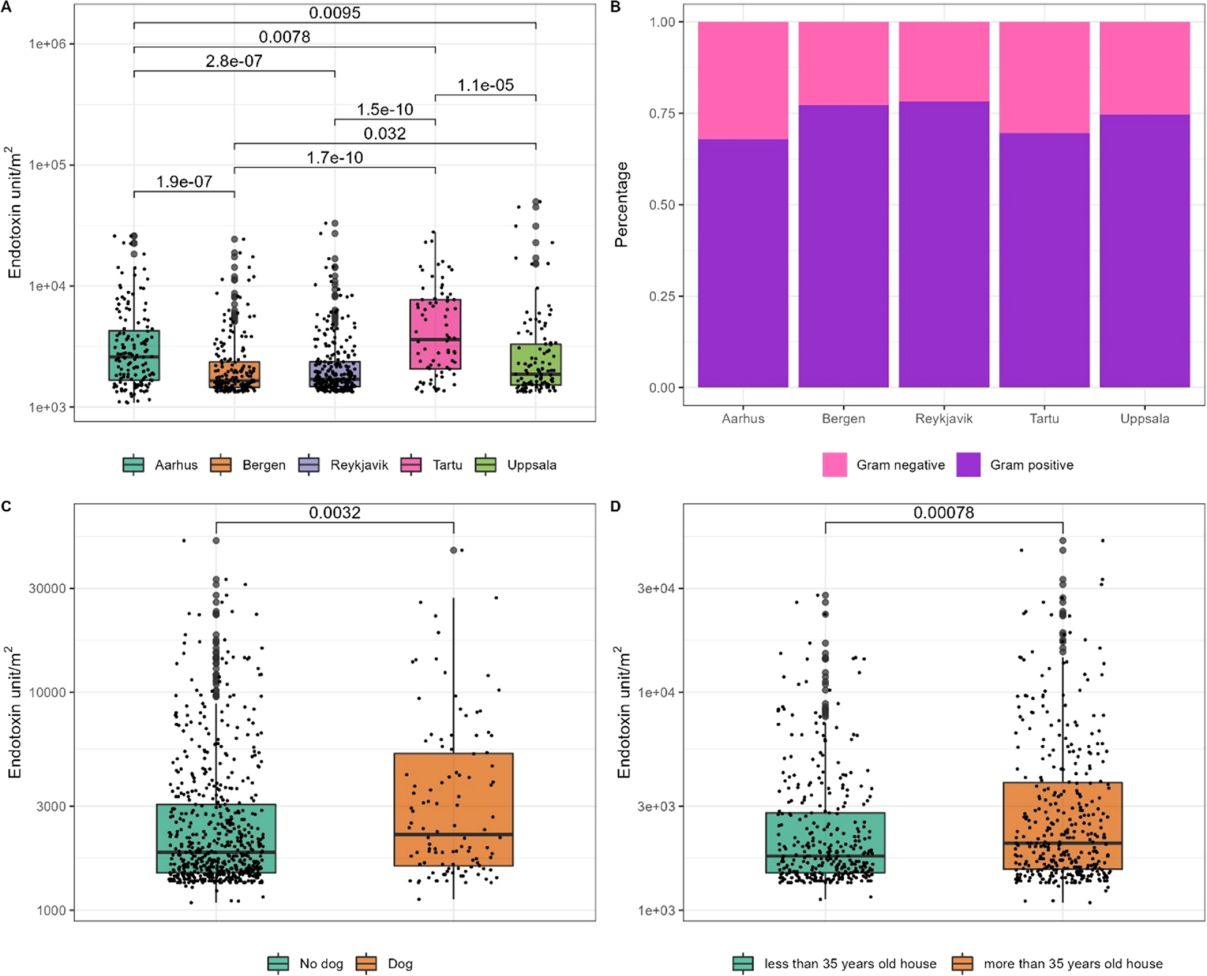
(A) Boxplot of endotoxin result of five cities’ households; (B) relative abundance of Gram-negative and Gram-positive bacteria of the five cities; (C) boxplot of endotoxin measurements of dog in bedroom (no vs yes); (D) boxplot of endotoxin result for older houses compared to more recently built houses. *P* values based on pairwise sample comparison in Wilcoxon signed-rank test. Only the significant pairwise comparison is shown.

Bergen households had a significantly lower endotoxin load than the other cities except Reykjavik households. Tartu households, on the other hand, had significantly higher endotoxin load compared to the other four cities’ households (Figure 5A). A higher relative abundance of Gram-negative bacteria was found in the Tartu and Aarhus households than in the other cities (Figure 5B). We found endotoxin concentration to be significantly correlated with the relative abundance of the three most abundant Gram-negative phyla, *Proteobacteria* (*r* = 0.32) followed by *Bacteroidota* (*r* = 0.17) and *Myxococcota* (*r* = 0.071) (Supporting Figure 8). Based on the Wilcoxon signed-rank test, there was a significant increase in endotoxin concentration in the indoor dust when dogs were allowed inside the bedroom and in older house groups compared to newer buildings (Figure 5C,D).

### 3.7 Meteorological Data

**Figure 6:**
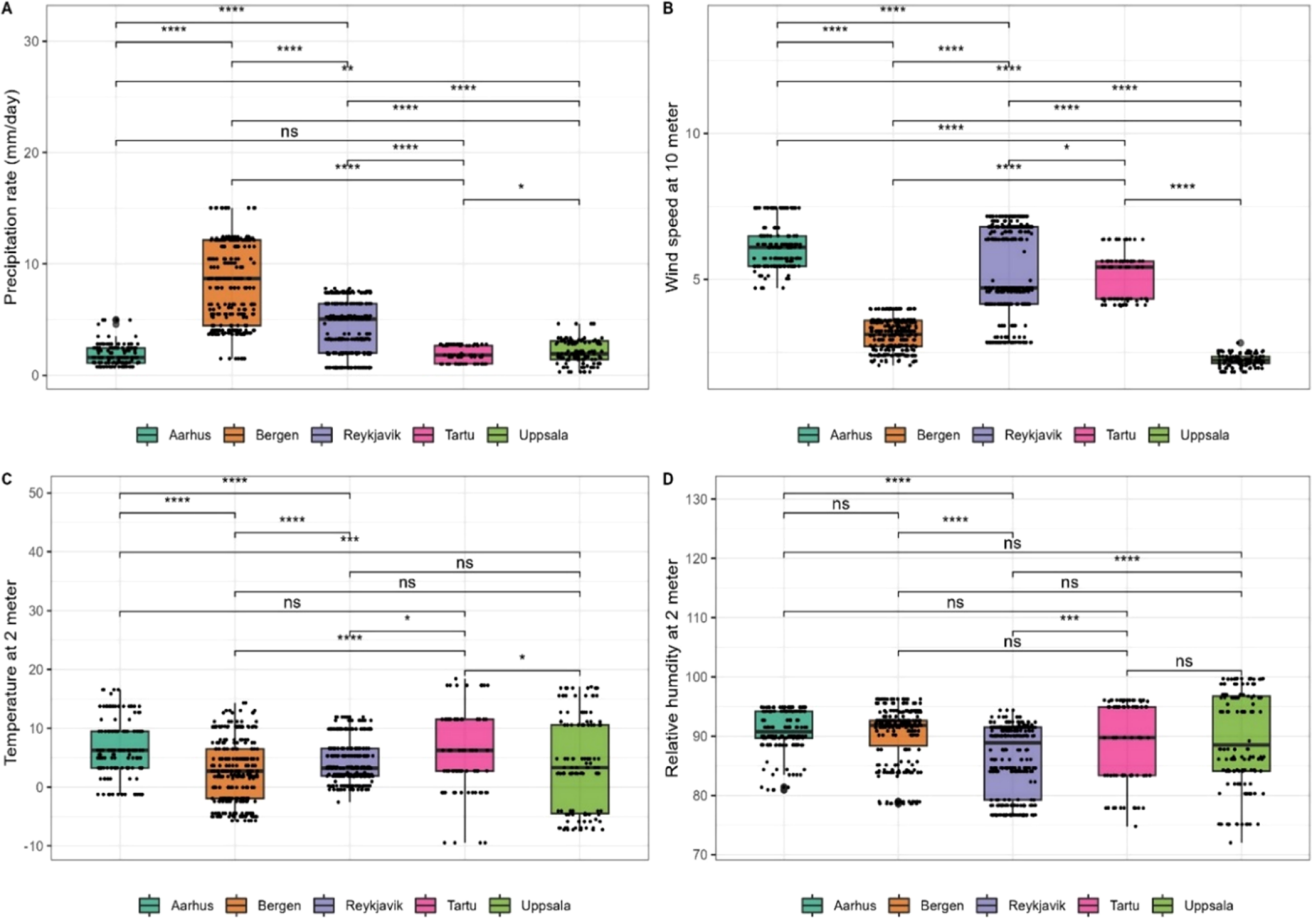
Box plots of monthly average meteorological data for the five cities during sampling of settled indoor airborne dust: (A) precipitation rate (mm/day), (B) wind speed (mm/s), (C) temperature (°C), and (D) relative humidity (%). An asterisk (*) indicates a significant pairwise comparison (*P* value ≤ 0.05). The greater the number of asterisks, the lower the *P* value. Nonsignificant pairwise comparison between cities, indicated by (ns).

The average monthly precipitation rate during sampling of settled indoor dust was significantly higher in Bergen compared to the other cities. There was no statistically significant difference between precipitation rates in Aarhus and Tartu (Figure 6A). Wind speed and temperature were significantly higher in Aarhus and in Tartu compared to other cities (Figure 6B,C). The relative humidity was not significantly different between the cities except for Reykjavik, which had significantly lower relative humidity than other cities (Figure 6D).

The precipitation rate was negatively correlated with indoor air bacterial diversity, bacterial load, and endotoxin load. On the other hand, wind speed was positively correlated with both bacterial and endotoxin load. Bacterial diversity was found to be positively correlated with temperature and negatively correlated with relative humidity (Table 2). Scatter plots of the correlation coefficient between the meteorological data and indoor air bacterial diversity, bacterial load, and endotoxin load can be found in Supporting Figures 9–11.

**Table 2:** Spearman Rank Correlation Coefficients between the Meteorological Data and Indoor Airborne Bacterial Measurement

	Shannon index		Bacterial load		Endotoxin load	
	*r* value	*P* value	*r* value	*r* value	*P* value	*r* value
precipitation rate	–0.16	<0.001	–0.13	<0.001	–0.19	<0.001
wind speed	–0.003	0.91	0.11	<0.001	0.12	0.001
temperature	0.14	<0.001	0.08	0.008	–0.02	0.9
relative humidity	–0.10	0.001	–0.02	0.49	0.06	0.1

## 4 Discussion

We investigated the role of occupants and indoor determinants on the bacterial microbiome of airborne indoor dust from 1038 households in five Nordic cities and showed that the variation in the airborne bacterial community is associated with six environmental determinants: geographical location, occupant’s age, number of occupants, presence of a dog, cleaning, and house age. Furthermore, we found a meteorological characteristic to be correlated with the indoor airborne bacterial community. Here we emphasize precipitation, which was negatively correlated with the diversity and the load of the indoor airborne bacterial community.

### 4.1 Sources of Indoor Airborne Microbiome

In all five cities, the human body microbiome was the major contributor to the indoor bacterial microbiome. Indoor dust samples were dominated by Gram-positive bacteria, including a subset of bacterial genera known to be associated with humans (*Staphylococcus*, *Streptococcus*, *Micrococcus*, *Corynebacterium*, and *Lactobacillus*). Not surprisingly, many bacterial genera could be traced back to the human skin, although gut and oral environments also contribute.^10,15,30,31^ Outdoor bacteria for example *Sphingomonas*, *Rhodococcus*, and *Arthrobacter* contributed to the composition of the indoor microbiome in all cities.^32−35^ These bacteria may enter houses via windows and doors or could be transferred from shoes onto floors and carpets and then become resuspended in indoor air. This is in line with previous studies that showed that both the occupants and outdoor environments are the major sources of microorganisms found indoors.^10,11^

The taxa from outdoor sources such as *Sphingomonas*, *Rhodococcus*, and *Arthrobacter* were more abundant in Tartu and Aarhus household compared to other cities, which might explain the higher bacterial load and bacterial diversity in these two cities’ households as outdoor bacteria are among key sources of bacteria in indoor. An increase in the relative abundance of Gram-negative bacterial taxa that mainly originate from outdoor sources such as *Protobacteria*, *Acinetobacter*, and *Skermanella*(16,36) and the increase in the bacterial load might together explain the higher endotoxin (i.e., a cell component of Gram-negative bacteria) load in Tartu and Aarhus compared to other cities’ households that were characterized by fewer outdoor bacterial taxa.

The weaker link between endotoxin and indoor characteristics, compared to bacterial diversity and load observed throughout the current study, may be due to the dominance of Gram-positive bacteria from human skin indoors, which lack endotoxin. Factors related to humans and their behavior such as cleaning frequency, number of occupants, and occupants’ age explain variations in bacterial diversity and load indoors but not endotoxin levels. In contrast, outdoor bacteria, rich in Gram-negative bacteria (containing endotoxin), contribute to higher endotoxin levels, influenced by outdoor activities like owning a dog.

### 4.2 Geographical Location and Meteorological Data

The meteorological factors, which are known to impact outdoor microbial communities,^37^ might explain why there are different amounts of outdoor bacterial taxa in households located in different cities. In a previous study using wipes from the external surfaces of approximately 1200 households located across the United States, the authors found continental-scale distributions of the outdoor bacteria and suggested that change could be related to climate factors.^38^ In the current study, Tartu and Aarhus were characterized by lower precipitation rates and higher wind speeds compared to other cities, while the temperature and relative humidity were within similar ranges. Fu et al. recently reported that the microbial community inside a building is affected by different outdoor environmental factors, such as geographical characteristics, precipitation, and relative humidity.^39^ In the current study, wind speed and temperature were positively correlated with bacterial load and diversity, while precipitation was negatively correlated with bacterial diversity, bacterial load, and endotoxin load. High wind speeds might have increased the outdoor bacterial concentrations and thus the amount of outdoor bacterial taxa that infiltrated from outdoor air into indoor air in Aarhus and Tartu. Thus, indoor bacterial diversity and load were both higher in these two cities. Yafeng et al. measured the outdoor and indoor PM2.5 (Particulate Matter 2.5) concentrations, which is an important carrier medium for bacteria. The author found the indoor infiltration rate of PM2.5 to be positively correlated with outdoor wind speed and temperature.^40,41^ Bergen was characterized with higher precipitation than other cities which might explain the lower abundance of outdoor bacterial taxa in Bergen households compared to the households in other cities through decreased infiltration of outdoor air particles. Rainfall is known to scavenge atmospheric particles, including bacteria, and transport them to the ground in a process known as “wet deposition,” which increases with rainfall intensity.^42^ However, the impact of raindrops on various surfaces on the ground might triggers the emission of surface-associated bacteria into the atmosphere,^19,43^ which likely depends on the type of source environments.^44^ So, it is likely the combinations of these two processes that will determine the concentration and type of airborne bacteria in outdoor air. Huffman et al. found that in a forest ecosystem the concentration of airborne biological particles increased significantly due to rainfall.^45^ Tian et al.^46^ established that the concentration of coarse aerosol particles (>2.5 μm in diameter) in urban environments was reduced by rain,^40,46^ which fits well with our observation that heavier rainfall is associated with reduced outdoor bacteria indoors. In addition, rainfall was found to alter the composition of airborne bacterial community at a suburban site with an increase in the relative abundance of *Actinobacteria* and a decrease in the relative abundance of *Proteobacteria*, which matches the bacterial profile of Bergen, a city known for heavy rainfall.^19^

Exposure to a variety of microorganisms has been inversely associated with the risk of developing asthma and atopy.^8,47−49^ With this in mind, Kirjavainen et al.^9^ found that the protective “farm-like” microbiota against asthma and atopy had a higher abundance of outdoor-associated bacterial taxa, including *Sphingobacetria* and *Alphaproteobacteria* bacteria. These taxa were less abundant in Bergen compared to Aarhus and Tartu which might be related to higher precipitation and lower wind speeds that hinder the outdoor taxa to enter the homes in Bergen. The intensity of precipitation is expected to intensify with global warming,^50,51^ and if our assumption is correct, this will increase wet deposition of outdoor particulates and particles associated with bacteria. As a result, fewer outdoor bacteria will contribute to the indoor microbiome and the intensity of exposures to environmental bacteria and endotoxins will decrease, with possible negative consequences for the development and maintenance of a tolerogenic immune status.^52^

### 4.3 Occupants’ Age

Human skin microbiota is considered a principal source of indoor airborne bacteria.^16^ The occupant’s age was for the first time associated with an increase in bacterial diversity, a reduction in bacterial load, and a change in the composition of the bacterial community. However, we are aware that in the current study, only the age of the participant in the ECRHS study was known, while the age of other occupants who used the same bedroom where settled dust samples were collected was unknown.

The human skin microbiome undergoes age-associated changes that reflect underlying age-related alteration in the cutaneous structure and the physiological function of the skin.^53^ Several studies have shown that bacterial species richness and diversity increase gradually with advancing age.^53−55^ Howard et al. investigated the skin microbiome of 158 females aged 20–74 years old and showed that bacterial diversity increased with age. The authors also found a change in the relative abundance of several bacterial taxa between different age groups.^55^ This supports our ANCOM BC results showing that 16 bacterial genera were differentially abundant between the two age groups in the current study. The number of bacteria on the skin tends to decrease with age, which also supports the results of our study. According to Lyden et al. sebum secretion levels decrease with age. As sebum is rich in triglycerides and free fatty acids, this leads to a decline in nutrients and consequently to a decrease in bacterial numbers.^56^

### 4.4 Level of Occupancy

The number of occupants was associated with an increase in both bacterial diversity and richness. This is in line with previous results, demonstrating that high occupancy leads to an accumulation of human-associated microorganisms.^18,31,57^ The increase in bacterial diversity with increased human occupancy could be attributed to several causes: (1) bacteria emitted from occupants could differ between individuals^58,59^ and (2) a higher density will lead to enhanced activity and thus, more resuspension of floor dust particles, in addition to more transport of outdoor bacteria attached to clothes and shoes.^60,61^ In the present study, increasing occupancy was associated with an increase in bacterial load, which has also been shown in other studies.^10,16,62^ Qian et al., studying the microbiome of classrooms, found that the bacterial load was much higher during the active school days than during vacation.

### 4.5 Pets

While dogs significantly contributed to the indoor airborne bacterial community both in terms of composition and diversity, cats had little influence on the indoor microbiome. These results are consistent with previous reports on the impact of cats and dogs.^27,63,64^ An ANCOM BC analysis showed an increased abundance of several bacterial taxa we assume are either introduced by the dogs from the outdoor environment such as *Rhodococcus*, *Sphingomonas*, and *Arthrobacter*(16,32−34) or stem from the dogs’ own microbiome itself such as *Moraxella* and *Fusobacterium*, common members of a dog’s oral and gastrointestinal tract microbiome.^65,66^ This is in line with the finding of Dunn et al. who found that households with dogs had a higher relative abundance of bacterial taxa associated with dog microbiota.^12^ The presence of a dog in a household was also associated with a higher endotoxin load. This is in line with Fuertes et al. reporting that endotoxin concentration in air was associated with dogs but not with cats.^67^ In the current study, higher endotoxin loads might be explained by the dog’s own microbiota, such as *Moraxella* and *Fusobacterium*(65,66) These Gram-negative bacteria were found to be the most abundant taxa in the indoor air of the dog owners’ households, in addition to the Gram-negative environmental bacteria brought in by the dog from the outdoors.

### 4.6 Cleaning and Use of Disinfectant

Higher cleaning frequencies were associated with an increase in bacterial diversity and load of the indoor air. Cleaning might lead to resuspension of settled dust and air mixing, thus increasing the number of bacterial taxa collected by the EDCs. This could explain the increase in bacterial diversity and load associated with higher cleaning frequency. Thus, cleaning frequency is one of the behavioral choices that can influence our daily exposure to different bacterial species. Sordillo et al.^68^ observed that frequent cleaning increases muramic acid levels in indoor air, a component of Gram-positive bacteria’s cell wall, which is consistent with our current finding.

Use of cleaning and disinfecting agents was related to a lower abundance of several Gram-negative and Gram-positive taxa, especially when bleach (sodium hypochlorite) was used. Due to the lack of selectivity, common disinfection practices such as the use of sodium hypochlorite, would indiscriminately kill indoor air microorganisms.^1^ In the current study, samples were collected between 2011 and 2013. However, with the advent of the COVID-19 pandemic, the deployment of chemical disinfectants such as sodium hypochlorite has increased dramatically in various building environments.^69^ In a recent study conducted during the COVID-19 pandemic, regularly disinfecting school classrooms by spraying disinfectant and wiping indoor surfaces was found to reduce airborne bacteria. which is in line with our findings.^70^ Yet, it is necessary to conduct further research to understand the implications of altering the microbiome through intensified disinfection use on the health of individuals occupying the space.

### 4.7 House Age and Indoor Characteristics

In the present study, the age of the house was associated with an increase in bacterial diversity and richness. Previously, Kettleson et al.^27^ showed that an increase of fungal diversity was associated with the age of the building. They did not find the same association with bacterial diversity. However, the small sample size (*n* = 35) compared to our study (*n* = 1038) might have masked some of the patterns. An increase in bacterial diversity in older houses may be caused by leaky plumbing systems, providing access for bacteria that will be further transferred to the indoor air through the ventilation system.^10,17^ We have shown previously in ECRHSII that old buildings have more dampness and water leakages.^71^ In the present study, the differential abundance analysis showed that in older buildings, there was an increased abundance of bacterial taxa belonging mostly to aquatic environments, including *Friedmanniella*, *Ilumatobacter*, and *Microlunatus*.^72−75^ This implies that differences in the plumbing systems between old and new houses may affect the composition of the indoor airborne microbiome. Additionally, the age of the house was associated with an increased endotoxin load. Similarly, in a nationwide-scale study in the United States involving more than 800 homes, the authors found that the age of buildings was an important predictor of endotoxin concentration.^76^

Two indoor characteristics were associated with an increase in bacterial richness: type of bedroom floor and bedroom size. Maybe a bigger room size is accompanied by a bigger or larger window, which would increase the infiltration of outdoor bacterial taxa. Small-scale structured floors (i.e., rugs) contained more bacterial taxa than uniform surfaces such as fitted carpets. This was also reported by Weikl et al. who found that floor dust from rugs had a more diverse bacterial community composition than samples from carpets.^29^ The composition of airborne indoor bacterial communities showed a significant association with the presence of a rug in the bedroom. Studies report a significant increase in the abundance of three bacterial genera: *Sphingomonas*, *Pseudonocardia*, and *Friedmanniella*, which are also found outdoors.^16,77,78^ Most rugs are made of textile materials with high porosity, which facilitates the adherence of dust and organic compounds. In addition, the pores may also retain sufficient moisture.^79^ In combination, these factors might facilitate bacterial growth and persistence due to increased levels of organics and moisture.^1^

### 4.8 Ventilation

According to the ANOSIM test, the ventilation achieved by opening the window during sleep (natural ventilation) as well as the presence of wall vents, designed to supply fresh air to a residential building, in the bedroom (mechanical ventilation) were both associated with a minor but significant change in the composition of the bacterial community. This is in line with results published by Brągoszewska et al. who observed differences in bacterial community composition in dust samples collected from a mixed-use building with half of the offices using natural ventilation and the other half using a conventional mechanical ventilation system.^80^ Ventilation with wall vents was associated with lower bacterial diversity and richness. Kembel and colleagues found that mechanically ventilated rooms have less diverse bacterial communities than naturally ventilated rooms.^18^ A possible reason behind the lower bacterial diversity with mechanical ventilation compared to natural ventilation systems is the use of filters in mechanical ventilation system, which prevents fractions of the outdoor bacteria taxa and particulates from entering the building.^1^

### 4.9 Moisture and Mold

Condensation of water on windows during winter was associated with a decrease in the bacterial diversity. Condensation is a sign of an increase in moisture (air relative humidity) and is the result of relatively warm and moist air getting into contact with cold window surfaces.^81^ High relative humidity in the air reduces the aerosolization of microbes from indoor surfaces and thereby reduces dust resuspension into the air by occupant movements in comparison to low relative humidity, which increases the potential for aerosols to stay aloft longer and travel further.^48,82^ This might explain a decrease in bacterial diversity associated with condensation on windows during the winter.

Equilibrium relative humidity (ERH) is used to assess moisture at the material’s surface. When the ERH reaches certain threshold (e.g., 70% for wooden materials), the material surface may become a target for microbial growth allowing mold germination and proliferation.^17,83^ In the current study, visible mold was in fact associated with increased bacterial diversity. In line with our findings, Gupta et al. found that bacterial and fungal diversity values were positively correlated in the bed dust.^84^ In a study done in Finland that investigated 41 severely water-damaged homes with mold growth, the authors found that the bacterial diversity of house dust decreased significantly after the water damage was fixed.^85^ This shows that there is a link between excessive surface moisture and an increase in the number of bacteria and fungi in indoor air.

### 4.10 Implications, Strengths, and Limitations

In the current study, we utilized 1083 EDC samples from the bedrooms of private homes across northern Europe. The large size of the samples enabled robust statistical comparisons to be made, resulting in reliable information about the factors that influence indoor microbiome compared to studies that have been limited to single geographical sites and small sample sizes. We observed that the indoor bacterial microbiome differed substantially by geographical location, and we conclude that the difference in the abundance of outdoor bacteria in the households may be due to different weather events, especially the wind speed and the precipitation. We speculate that future predicted increase in precipitation rates due to global warming could impact our indoor bacterial exposure and might have negative consequences for our immune system. Our study was limited by not having simultaneous outdoor sampling. Therefore, further studies including both indoor and outdoor samples, as well as recordings of meteorological data may be necessary to provide a more complete understanding of the effects of weather on the contribution of outdoor bacterial taxa to the indoors. Another limitation of the current study is that we lacked information on land use which could, in combination with metrological factors, affect the composition of indoor microbiome.^86^

Age of the occupant of the homes was associated with higher diversity but lower microbial load. We suggest that this is due to the age-related changes in skin microbiome. Furthermore, our results suggest that general lifestyle choices such as the number of occupants, types of pets, cleaning frequency of the household, and use of chemical disinfectants impact the indoor microbiome. Thus, the presence of a dog increases, whereas the use of disinfectants decreases microbial exposure. The use of disinfectants has increased dramatically since the COVID-19 pandemic, and our results lead us to conclude that it is urgent to study further the effects of excessive use of disinfectants on the indoor airborne bacterial community as it may have negative consequences on human health. In conclusion, our study identifies (1) several factors that may be subject to intervention to improve our indoor microbiome and (2) that further research to establish causality is urgently needed.

## Supporting Information Available

Methods; pairwise differential abundance analysis between the five cities’ households; results for univariate and multivariate regression analysis between environmental determinants and bacterial profiles; histograms of independent variables; differential abundant bacterial genera; correlation plots between the relative abundance; and scatter plots of the correlation coefficient between the indoor bacterial load (PDF)
