# Supplementary material for: Indoor Airborne Microbiome and Endotoxin: Meteorological Events and Occupant Characteristics Are Important Determinants
Source: Environ Sci Technol. 2023 Jul 31;57(32):11750–66. doi: 10.1021/acs.est.3c01616 (PMC10433529; doi:10.1021/acs.est.3c01616)
Supplement: Supplementary file 1 — es3c01616_si_001.pdf [file es3c01616_si_001.pdf]

Supporting Information for Indoor Airborne Microbiome and Endotoxin: Meteorological Events and Occupant Characteristics are Important Determinants

Hesham Amin [1]*, Tina Šantl-Temkiv [2], Christine Cramer [3,4], Kai Finster [2], Francisco Gomez Real [1] , Thorarinn Gislason [5], Mathias Holm [6], Christer Janson [7,8], Nils Oskar Jõgi [1], Rain Jogi [9], Andrei Malinovschi [8], Ian P.G. Marshall [2], Lars Modig [10], Dan Norbäck [11], Rajesh Shigdel [1], Torben Sigsgaard [3], Cecilie Svanes [12,13], Hulda Thorarinsdottir [14], Inge M. Wouters [15], Vivi Schlünssen [3], Randi J. Bertelsen [1†]

[1] Department of Clinical Science, University of Bergen, 5021 Bergen, Norway.

[2] Section for Microbiology, Department of Biology, Aarhus University, 8000 Aarhus, Denmark.

[3] Department of Public Health, Environment, Work and Health, Danish Ramazzini Center, Aarhus University, Aarhus, Denmark.

[4] Department of Occupational Medicine, Danish Ramazzini Center, Aarhus University Hospital, 8000 Aarhus, Denmark

[5] Faculty of Medicine, University of Iceland, 102 Reykjavík, Iceland.

[6] Department of Occupational and Environmental Medicine, University of Gothenburg, 405 30 Gothenburg, Sweden.

[7] Department of Medical Sciences: Respiratory, Allergy, Sleep Research, Uppsala University, 751 85 Uppsala, Sweden.

[8] Department of Medical Sciences: Clinical Physiology, Uppsala University, 751 85 Uppsala, Sweden.

[9] Tartu University Hospital, Lung Clinic, 50406 Tartu, Estonia.

[10] Division of Occupational and Environmental Medicine, Department of Public Health and Clinical Medicine, Umeå University, 901 87 Umeå, Sweden.

[11] Department of Medical Sciences, Occupational and Environmental Medicine, Uppsala University, 751 85 Uppsala, Sweden.

[12] Department of Occupational Medicine, Haukeland University Hospital, 5053 Bergen, Norway.

[13] Centre for International Health, University of Bergen Department of Global Public Health and Primary Care, 5009 Bergen, Norway.

[14] Department of Anesthesia and Intensive Care, Landspitali University Hospital, 101 Reykjavik, Iceland.

[15] Institute for Risk Assessment Sciences, Faculty of Veterinary Medicine, Utrecht University, 3584 CS Utrecht, The Netherlands.

† Senior authorship.

* Correspondence**:**
Hesham Amin, [1]Department of Clinical Science, University of Bergen, Bergen, Norway.

Hesham.amin@uib.no.

Number of pages: 32

Number of the supplementary figures: 11

Number of the supplementary tables: 14

Number of the supplementary method sections: 4

Supplementary figures

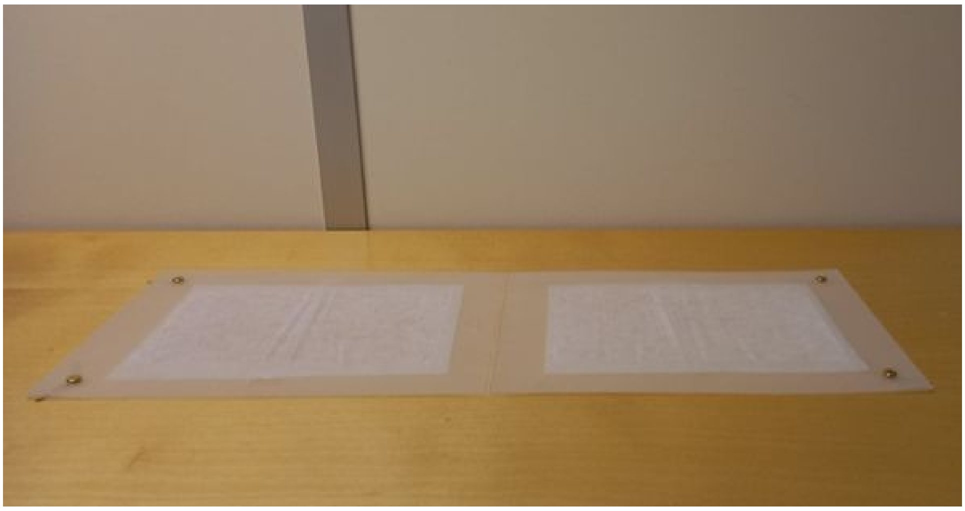

Supplementary figure 1: The EDC sampler is composed of two electrostatic cloths placed in a plastic folder that is left open in a horizontal position for 14 days to allow dust to settle. The folder was kept closed before and after sampling and during transport and storag

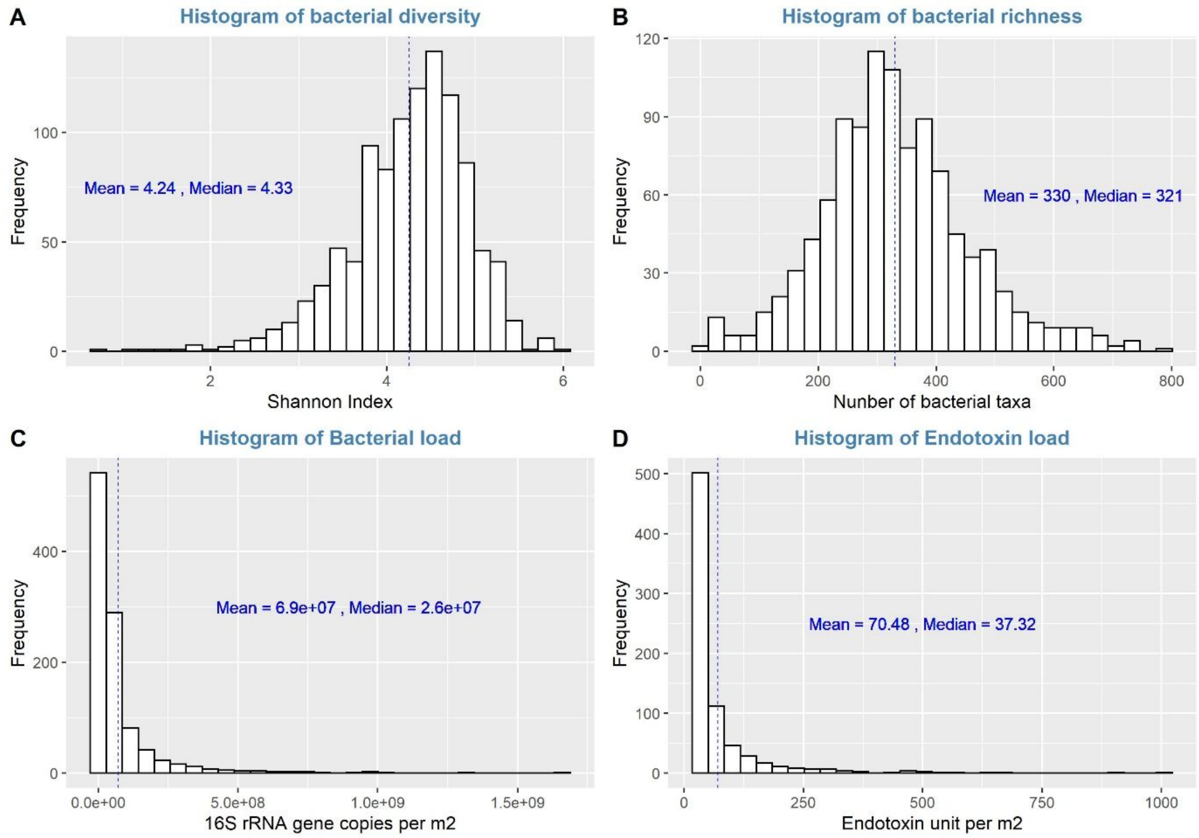

Supplementary figure 2: Histograms of independent variables A) Shannon index B) Number of bacterial taxa, C) 16S rRNA gene copies per m^2 of EDC, D) Endotoxin unit per m^2 the mean and the median were included inside the plot

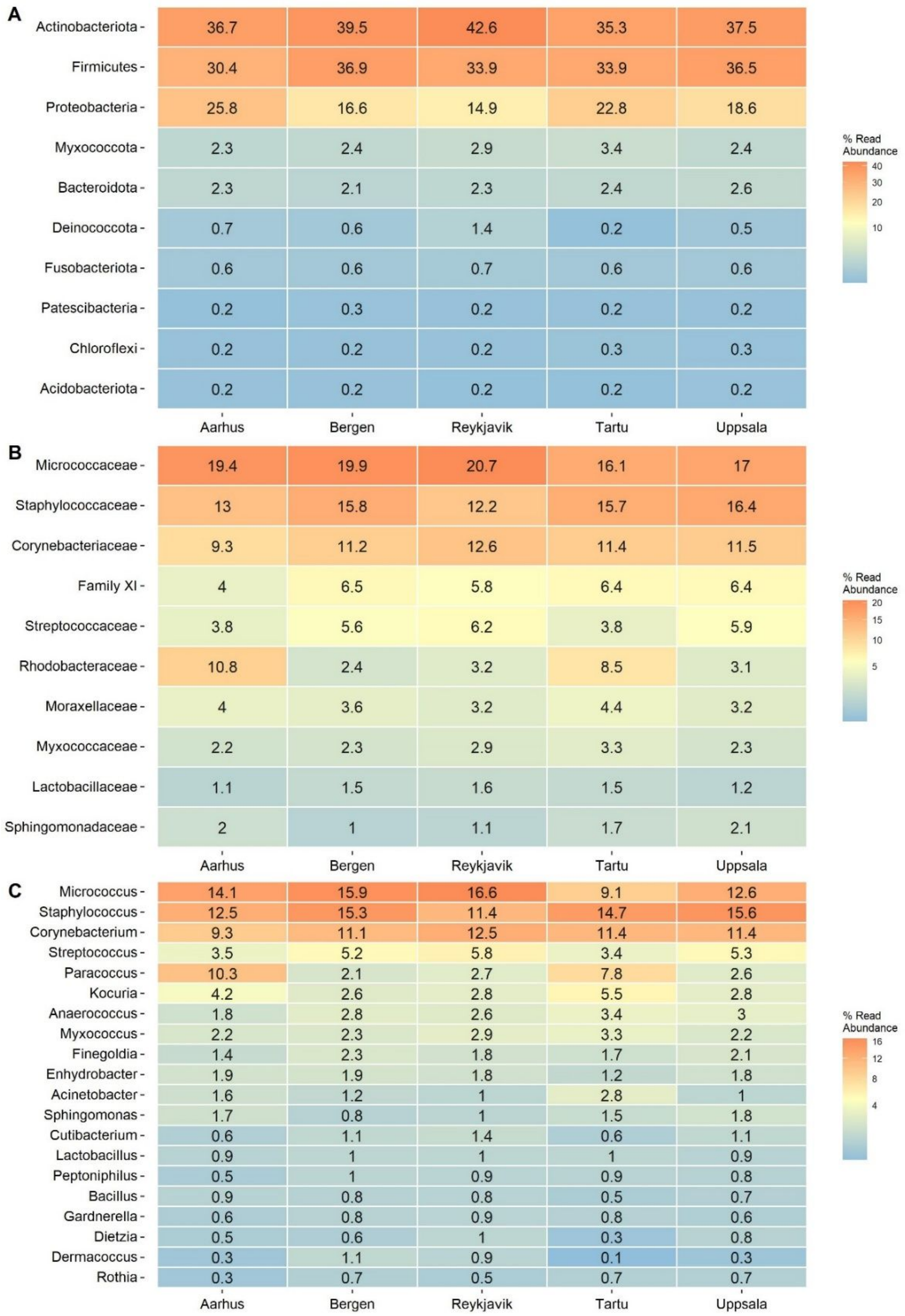

Supplementary figure 3: Heatmap including the community-level composition, the number indicating percentage (mean value of relative abundance) of bacterial taxa in the five cities. (A) top 10 bacterial phyla. (B) top 10 bacterial families (C) top 20 bacterial genera.

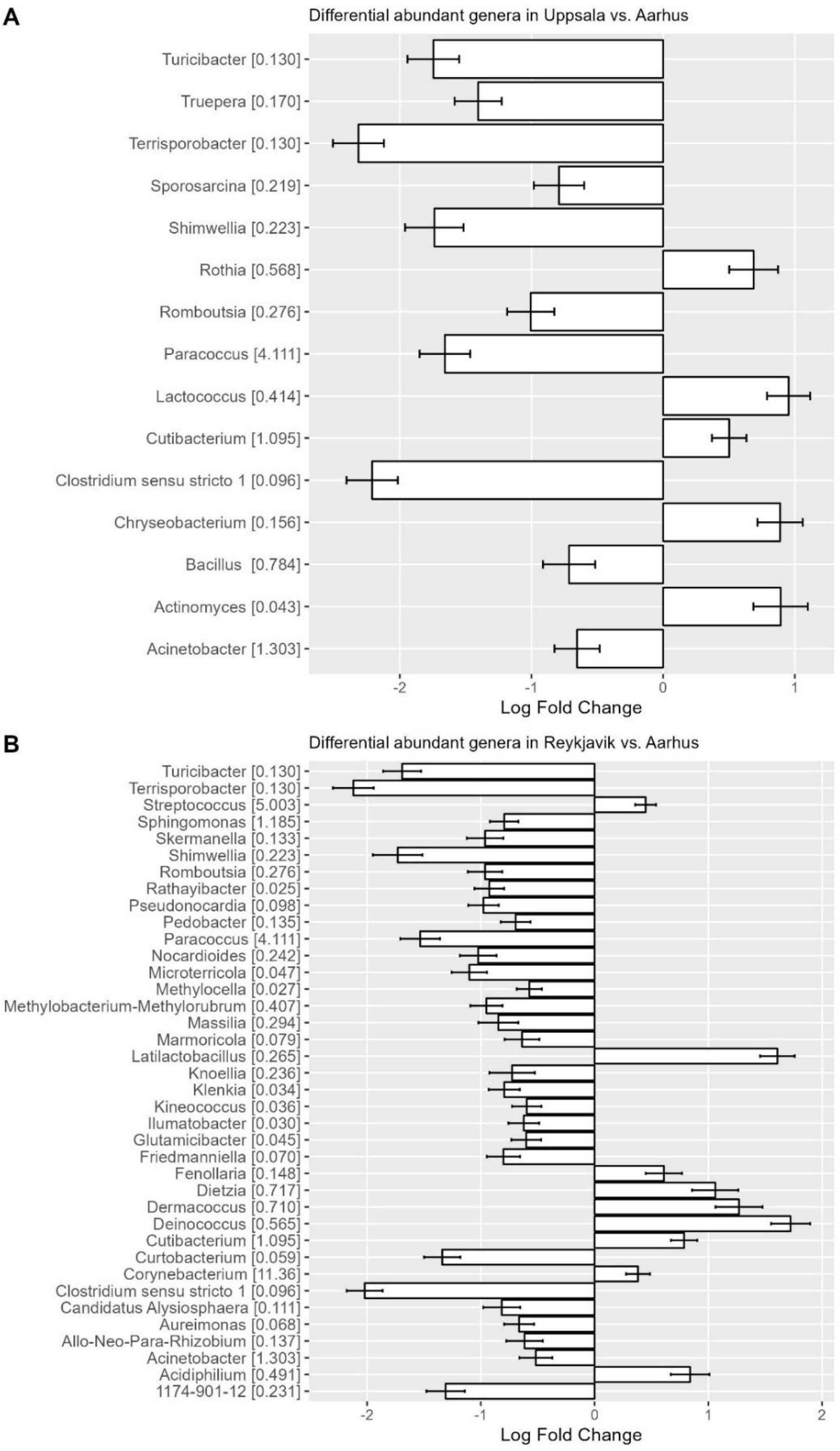

Supplementary figure 4: Differential abundant bacterial genera A) in Uppsala households compared to Aarhus households, B) in Reykjavik households compared to Aarhus households. Number in parentheses show relative abundance the bacterial genera in total number of the samples

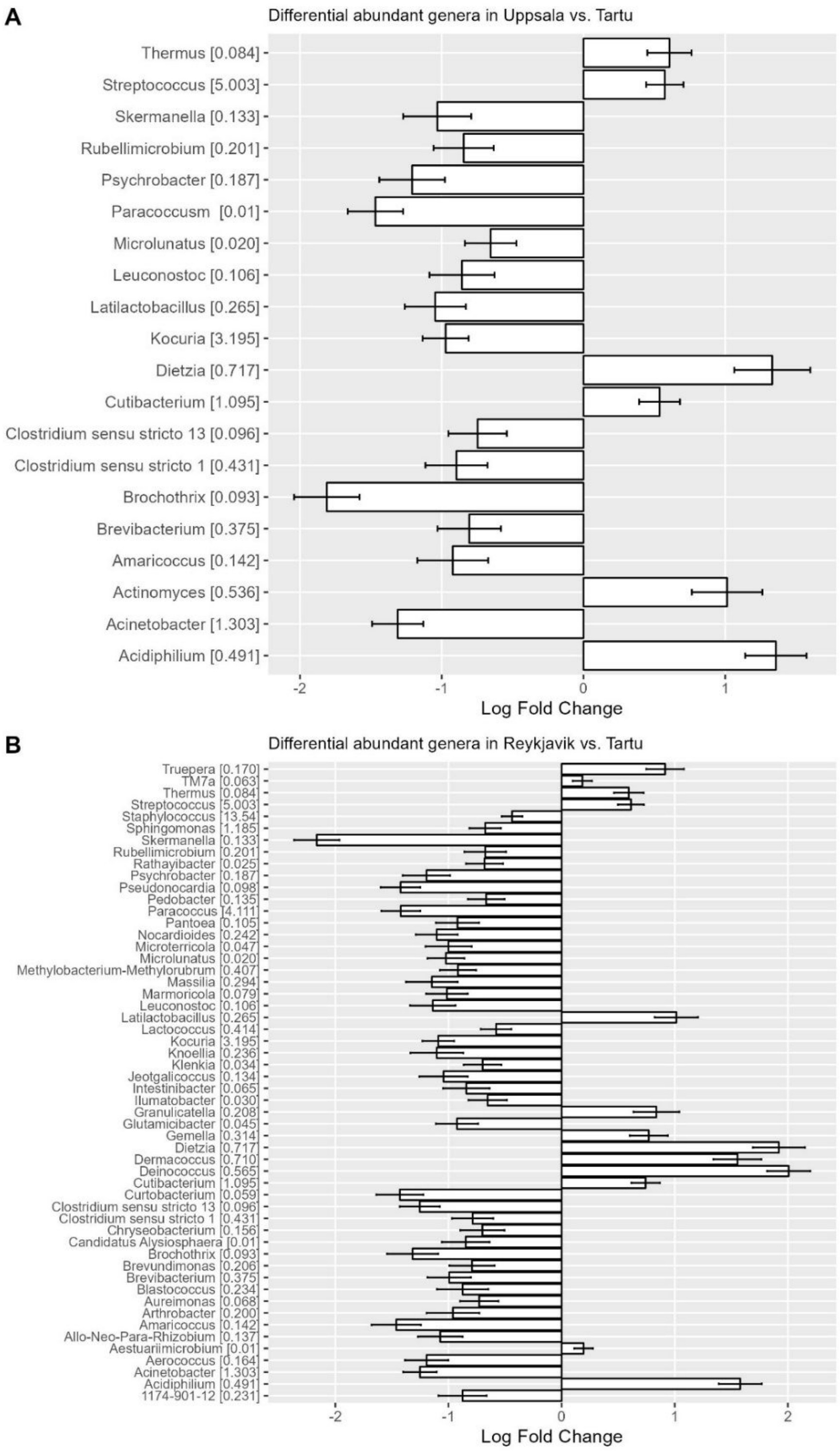

Supplementary figure 5: Differential abundant bacterial genera A) in Uppsala households compared to Tartu households, B) in Reykjavik households compared to Tartu households. Number in parentheses show relative abundance the bacterial genera in total number of the sample.

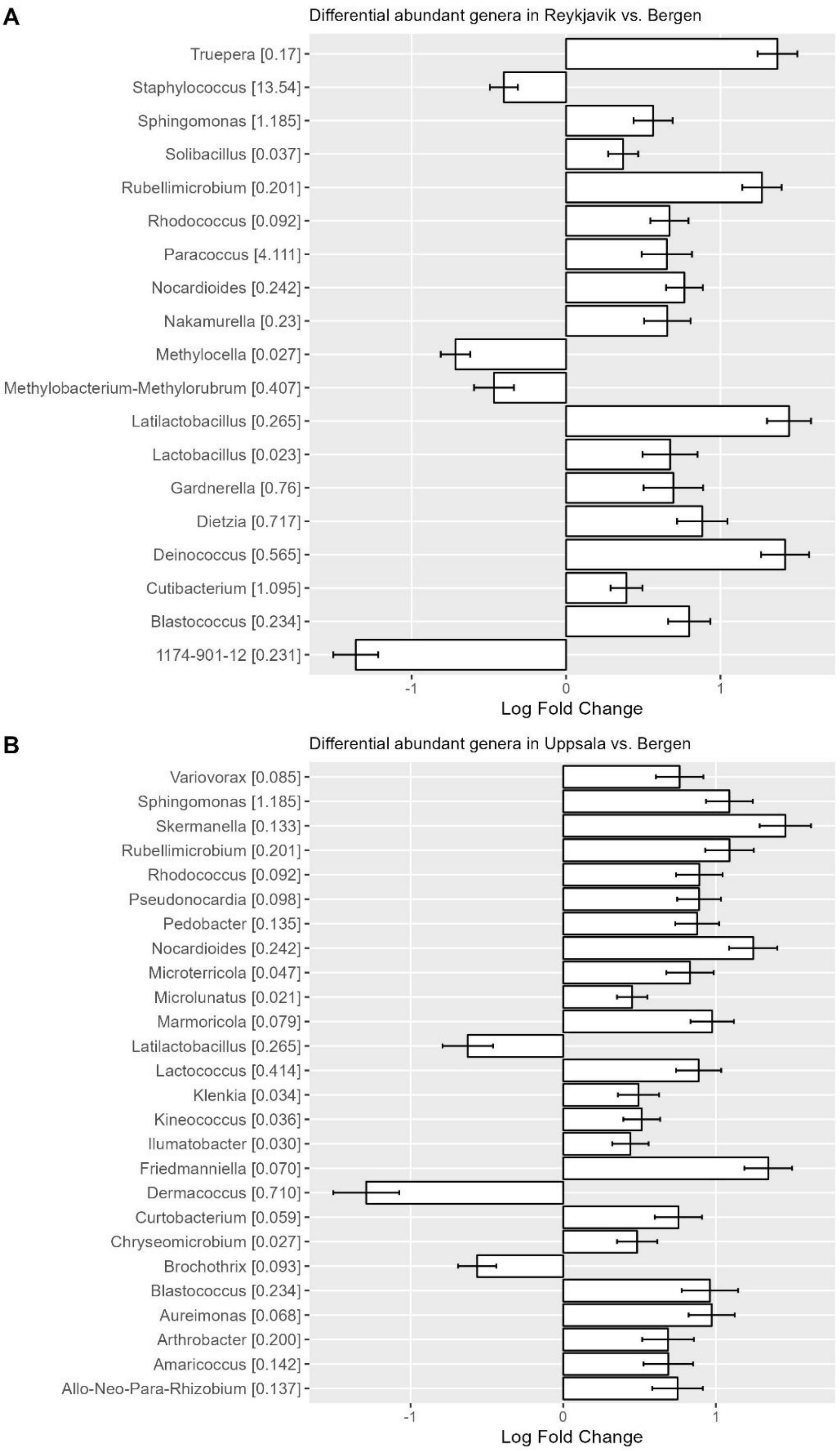

Supplementary figure 6: Differential abundant bacterial genera A) in Uppsala households compared to Bergen households, B) in Reykjavik households compared to Bergen households. Number in parentheses show relative abundance the bacterial genera in total number of the samples

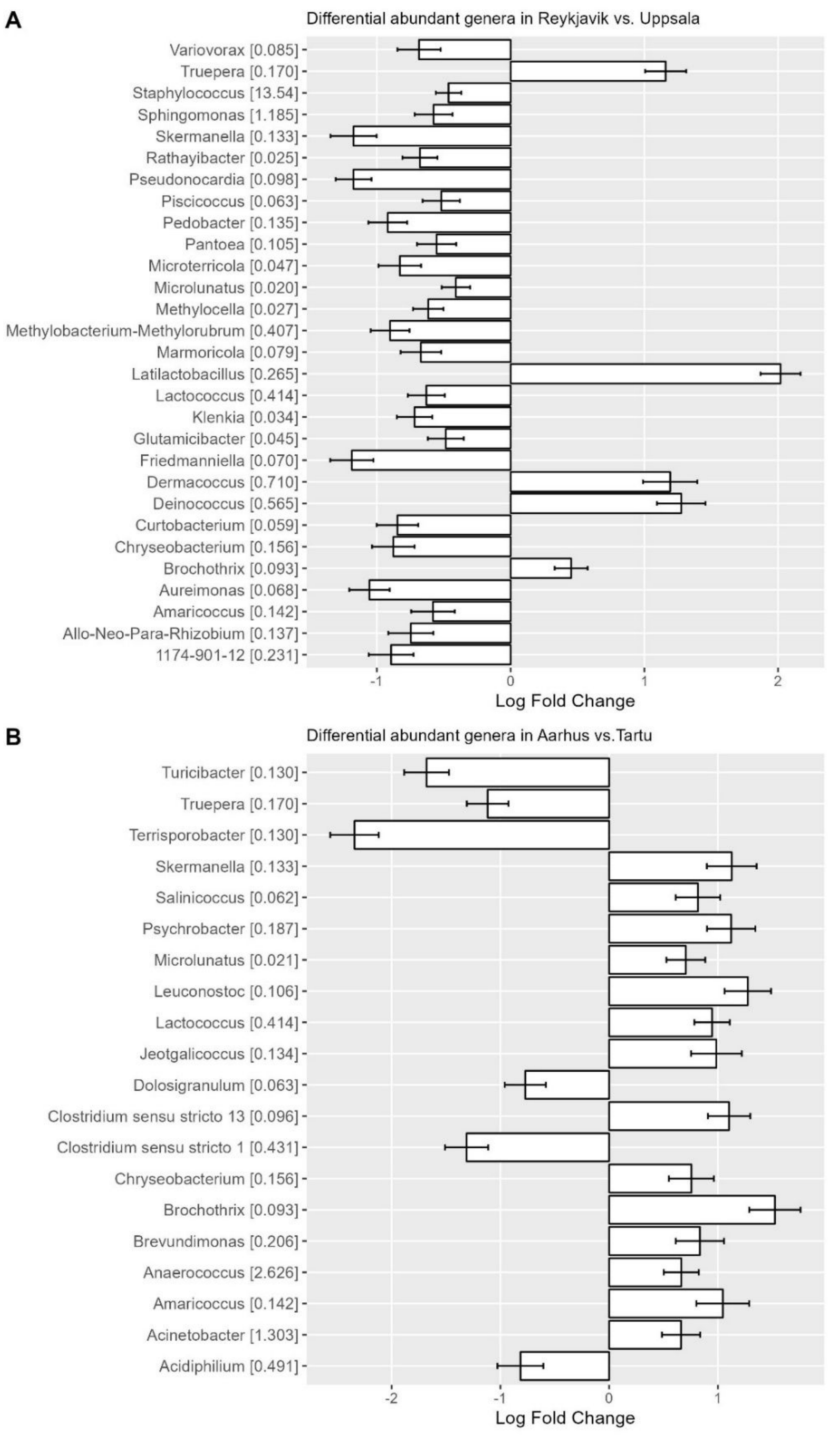

Supplementary figure 7: Differential abundant bacterial genera A) in Uppsala households compared to Reykjavik households, B) in Tartu households compared to Aarhus households. Number in parentheses show relative abundance the bacterial genera in total number of the sample

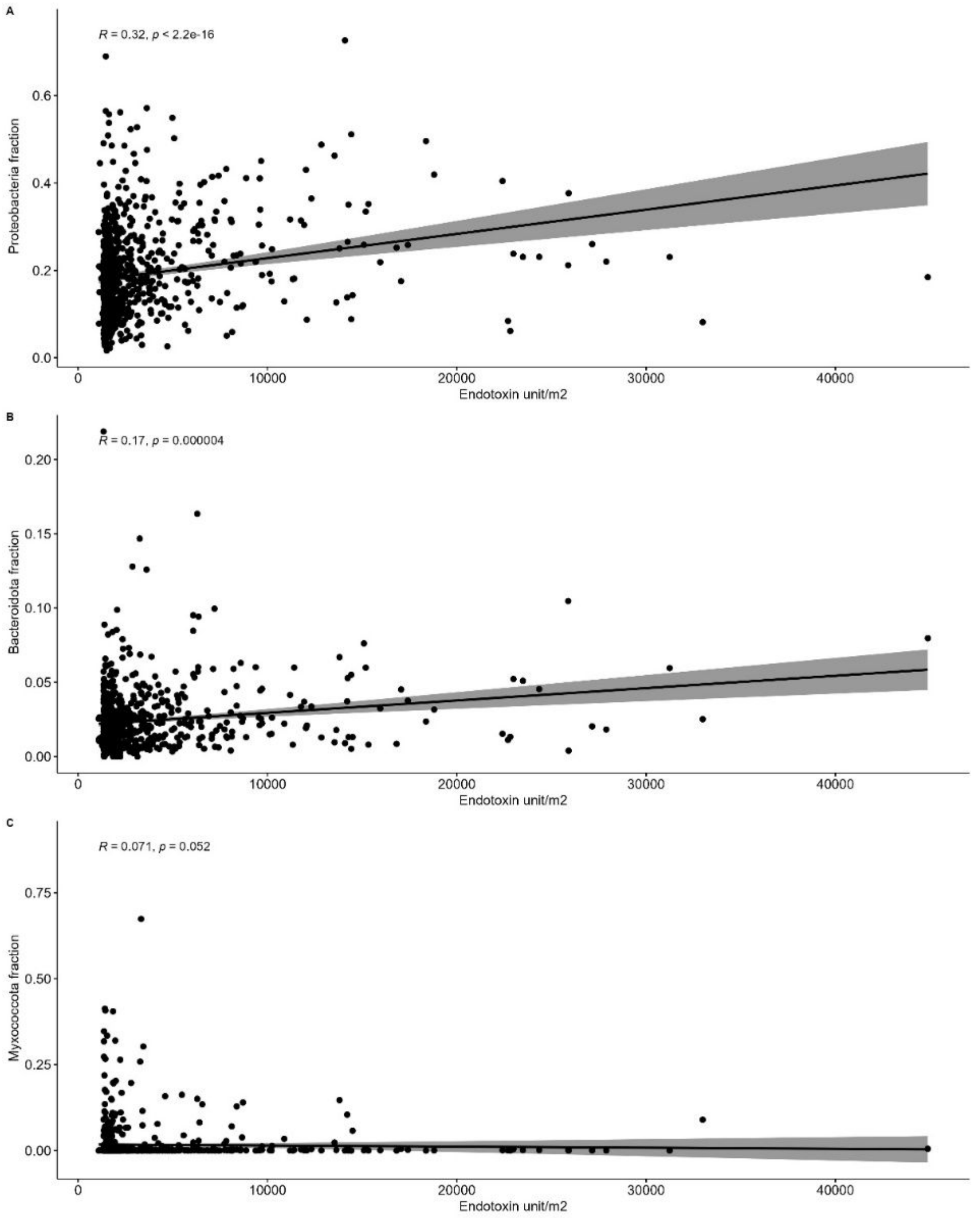

Supplementary figure 8: Correlation plots between the relative abundance (%) of A) Proteobacteria, B) Bacteroidota C) Myxococcota and endotoxin concentration.

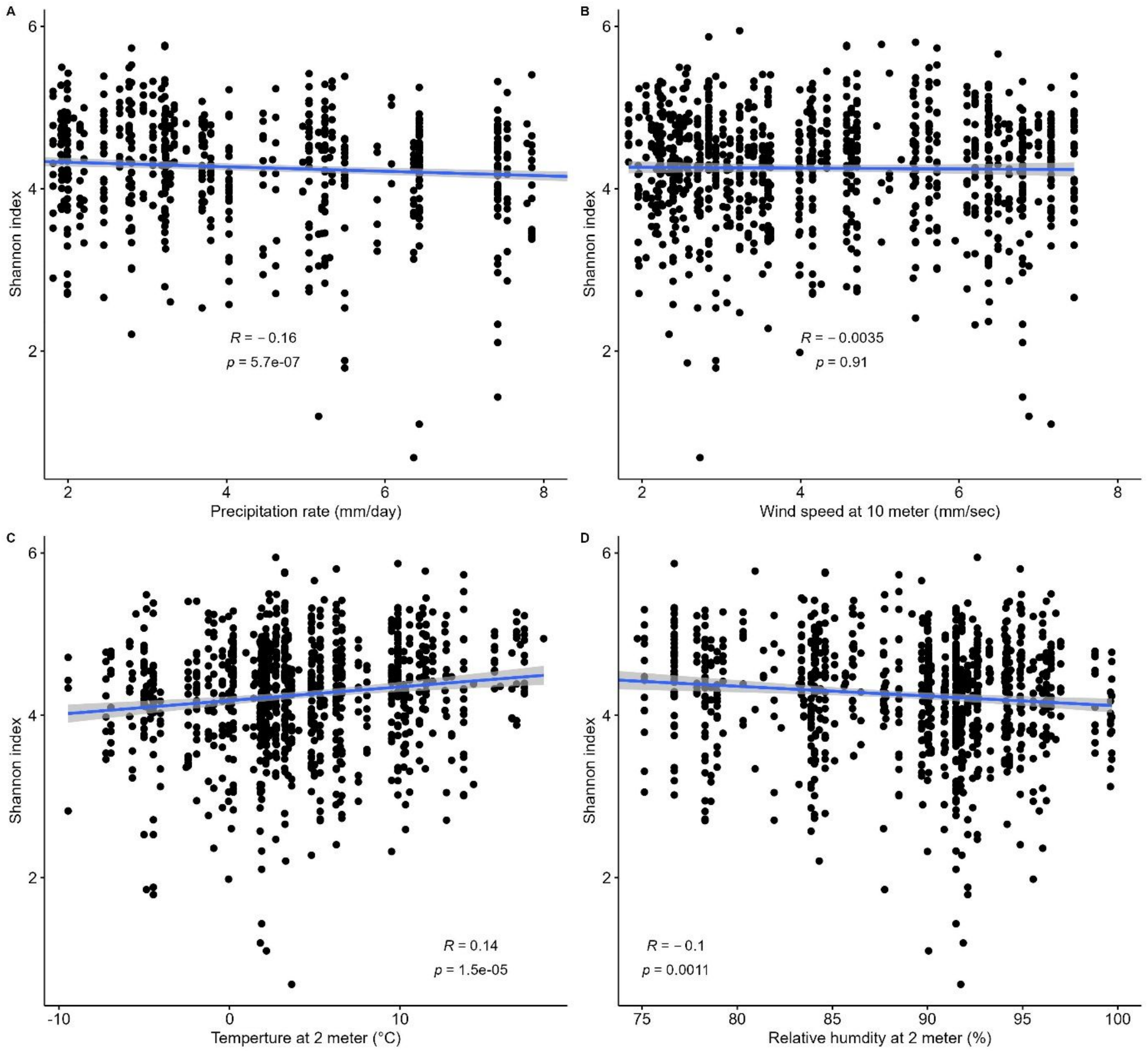

Supplementary figure 9: Scatter plots of the correlation coefficient between the indoor Shannon index (index of bacterial diversity) and the meteorological data A) precipitation rate (mm/day) B) Wind speed (mm/sec) C) temperature (°C) D) Relative humidity (%).

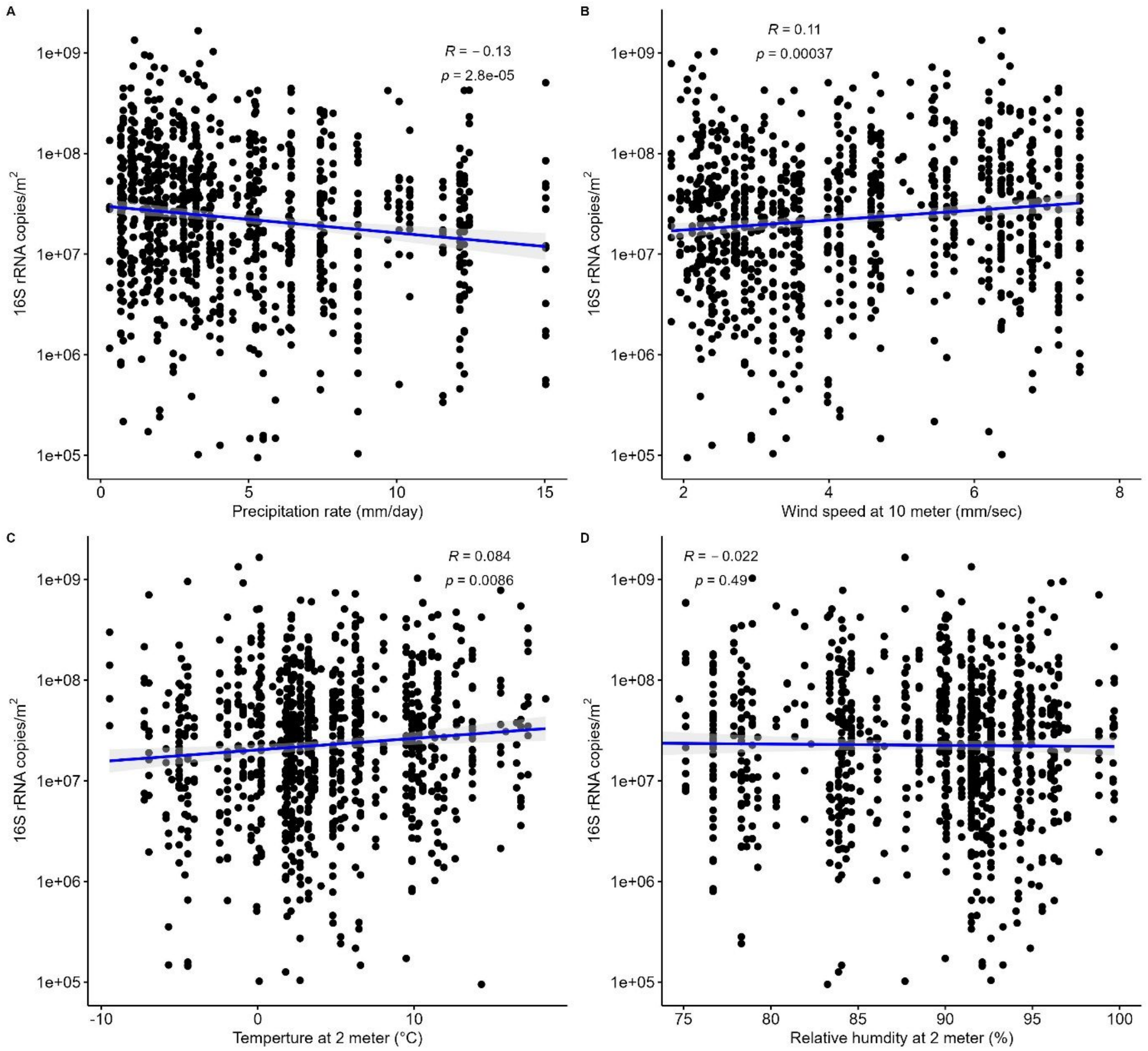

Supplementary figure 10: Scatter plots of the correlation coefficient between the indoor bacterial load (16S rRNA copies/m2) and the meteorological data A) precipitation rate (mm/day) B) Wind speed (mm/sec) C) temperature (°C) D) Relative humidity (%).

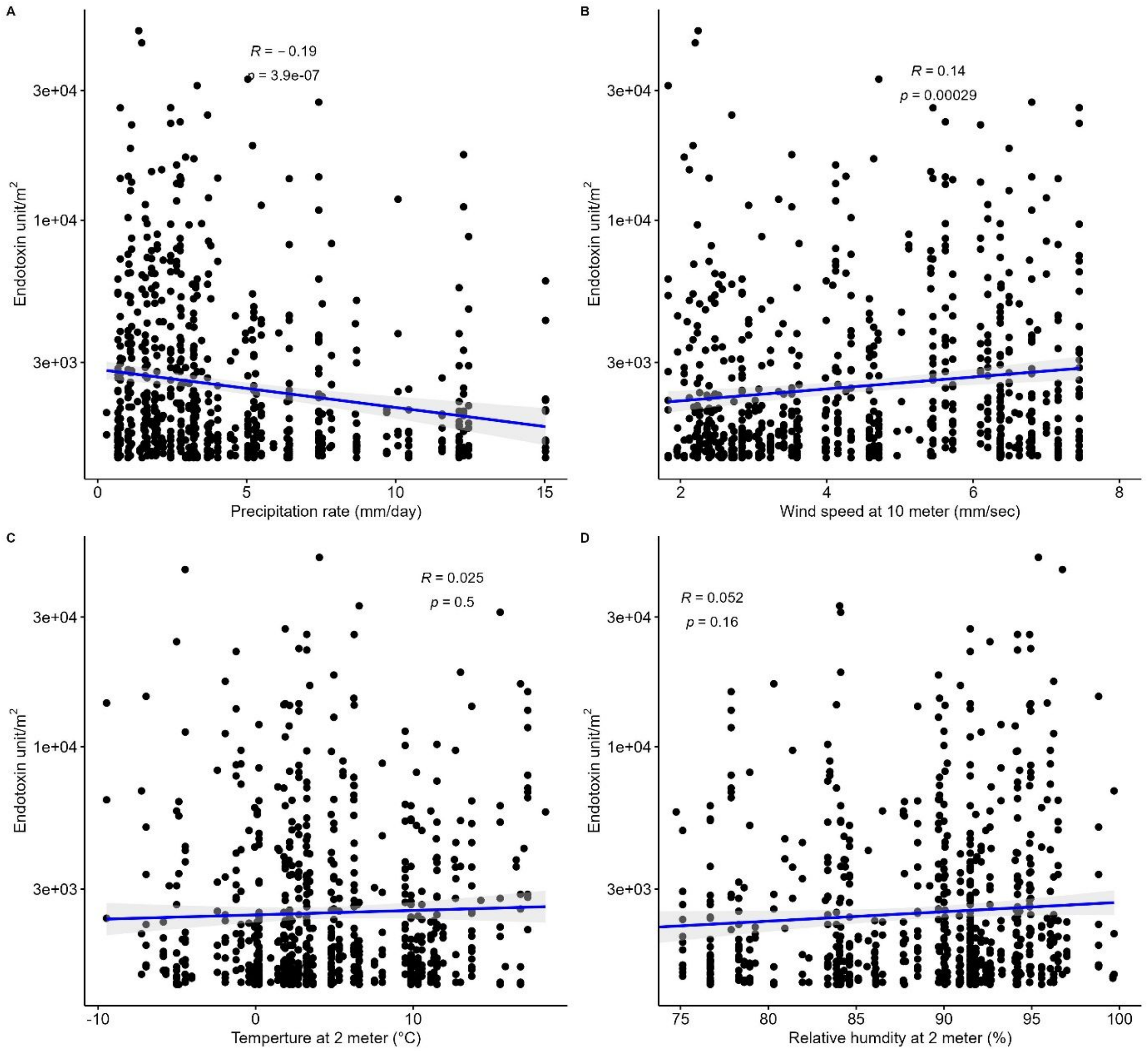

Supplementary figure 11: Scatter plots of the correlation coefficient between the indoor endotoxin load (endotoxin unit/m2) and the meteorological data A) precipitation rate (mm/day) B) Wind speed (mm/sec) C) temperature (°C) D) Relative humidity (%).

Supplementary tables

Supplementary Table 1: The relative abundance of Gram-positive and Gram-negative bacterial phyla across the five cities

Phylum	Gram stain	Aarhus	Bergen	Reykjavik	Tartu	Uppsala
**Actinobacteriota**	Positive	36.657	39.520	42.615	35.317	37.533
**Firmicutes**	Positive	30.423	36.947	33.899	33.907	36.453
**Proteobacteria**	Negative	25.827	16.616	14.921	22.807	18.591
**Myxococcota**	Negative	2.281	2.383	2.946	3.378	2.381
**Bacteroidota**	Negative	2.318	2.054	2.267	2.430	2.611
**Deinococcota**	Positive	0.657	0.575	1.427	0.183	0.457
**Fusobacteriota**	Negative	0.588	0.628	0.719	0.601	0.589
**Patescibacteria**	Positive	0.185	0.251	0.223	0.224	0.235
**Chloroflexi**	Negative	0.222	0.202	0.204	0.311	0.256
**Acidobacteriota**	Negative	0.218	0.240	0.164	0.194	0.249
**Campylobacterota**	Negative	0.070	0.128	0.131	0.094	0.103
**Verrucomicrobiota**	Negative	0.140	0.103	0.088	0.110	0.121
**Planctomycetota**	Negative	0.090	0.092	0.077	0.134	0.099
**Spirochaetota**	Negative	0.068	0.041	0.052	0.034	0.034
**Bdellovibrionota**	Negative	0.058	0.033	0.034	0.069	0.062
**Armatimonadota**	Negative	0.034	0.055	0.032	0.021	0.031
**Abditibacteriota**	Negative	0.048	0.021	0.043	0.037	0.037
**Desulfobacterota**	Positive	0.024	0.028	0.042	0.030	0.038
**Gemmatimonadota**	Negative	0.035	0.024	0.029	0.040	0.037
**Fibrobacterota**	Negative	0.007	0.006	0.015	0.006	0.005
**Nitrospirota**	Negative	0.006	0.006	0.010	0.015	0.011
**Dependentiae**	Negative	0.003	0.003	0.005	0.004	0.004
**Synergistota**	Negative	0.002	0.003	0.003	0.002	0.003
**Sumerlaeota**	Negative	0.002	0.004	0.001	0.002	0.004
**Elusimicrobiota**	Negative	0.002	0.002	0.002	0.001	0.002
**Other**	---	0.035	0.034	0.053	0.05	0.053

Supplementary table 2: Univariate quintile model estimates for bacterial load (16S rRNA/ m^2) of indoor airborne microbiome. All independent variables showed p value ≤ 0.25 in univariable quintile regression were used in multivariate quintile regression analysis.

Variable	16S rRNA/m^2	
	**B**	**p value**
**City (ref: Aarhus)**		
**Bergen**	-2.27e+07	0.001
**Reykjavik**	-1.38e+07	0.01
**Tartu**	1.58e+07	0.09
**Uppsala**	-7.80e+06	0.21
**Season winter vs summer**	3.60e+06	0.25
**Dog (No vs yes)**	1.07e+07	0.06
**Cat (No vs yes)**	1.52e+07	0.02
**No of occupants (1 vs ≥ 2)**	1.16e+07	0.003
**Occupant's age**	-9.70e+05	0.001
**Cleaning frequency (ref: < 1 day per week)**		
**1-3 days per week**	3.90e+06	0.24
**4-7 days per week**	1.41e+07	0.01
**Use of bleach (No vs Yes)**	-4.70e+06	0.17
**Use of ammonia (No vs Yes)**	-4.50e+06	0.31
**Window open at night (ref: Never)**		
**Sometimes**	3.00e+06	0.52
**All of the time**	-5.70e+06	0.1
**Kitchen fan use (ref: Never)**		
**Sometimes**	-1.00e+07	0.07
**All of the time**	-1.04e+07	0.05
**House age**	3.58e+04	0.55
**Mattress age**	-3.26e+05	0.25
**Central heating**	7.80e+06	0.01
**Electric heating**	-7.80e+06	0.01
**Ducted heating**	-3.80e+06	0.55
**Open coal heating**	-1.38e+07	0.001
**Radiators heating bedroom**	8.00e+06	0.01
**AC**	-1.19e+07	0.02
**Airbrick bedroom**	4.80e+06	0.32
**Fitted carpet**	-1.34e+07	0.01
**Rug bedroom**	-1.40e+06	0.69
**Damp spots bedroom**	2.34e+07	0.02
**Mould inside home**	1.41e+07	0.01
**Mould odour**	1.70e+07	0.03
**Condensation on window**	1.01e+07	0.01
**Water damage**	5.80e+06	0.11

Supplementary table 2: continue.

Variable	16S rRNA/m^2	
	B	p value
**No. of rooms (ref: One)**		
**Two**	7.10e+06	0.72
**Three or more**	-1.20e+07	0.47
**Floor level (ref: Ground floor)**		
**First floor**	1.51e+07	0.03
**Higher than first floor**	1.20e+07	0.08
**Bedroom size**	-8.46e+04	0.75
**Floor heating**	1.60e+06	0.76
**Bedroom wallpaper**	-4.50e+06	0.29
**Painted fiberglass**	-1.80e+06	0.71
**Wall vent**	-4.50e+06	0.16
**Ceiling exhaust**	-1.70e+06	0.82
**House type (ref: farmhouse)**		
**Detached house**	-5.50e+06	0.63
**Terraced house**	2.60e+06	0.84
**Apartment building**	-1.15e+07	0.32

Supplementary table 3: Quantile regression model estimates for bacterial load of indoor airborne microbiome expressed as 16S rRNA/m^2, include all independent variables showed p value ≤ 0.25 in univariate quantile regression.

**Variable**	**16S rRNA/m²**	
	**B**	**p value**
**City (ref: Aarhus)**		
**Bergen**	-2.35e+07	0.02
**Reykjavik**	-1.71e+07	0.009
**Tartu**	1.44e+07	0.18
**Uppsala**	-1.25e+07	0.08
**Season winter vs summer**	4.63e+06	0.14
**Dog (No vs yes)**	7.44e+06	0.22
**Cat (No vs yes)**	6.11e+07	0.27
**No of occupants (1 vs ≥ 2)**	1.12e+07	0.005
**Occupant's age**	-4.50e+05	0.05
**Cleaning frequency (ref: < 1 day per week)**		
**1-3 days per week**	2.74e+06	0.42
**4-7 days per week**	1.08e+07	0.05
**Use of bleach (No vs Yes)**	1.98e+06	0.54
**Window open at night (ref: Never)**		
**Sometimes**	7.57e+06	0.12
**All of the time**	1.14e+06	0.77
**Kitchen fan use (ref: Never)**		
**Sometimes**	-4.57e+06	0.35
**All of the time**	-5.04e+06	0.31
**Mattress age**	-1.44e+05	0.59
**Central heating**	-6.14e+06	0.43
**Electric heating**	3.29e+06	0.64
**Open coal heating**	-3.79e+06	0.53
**Radiators heating bedroom**	-2.80e+06	0.7
**AC**	-7.50e+06	0.21
**Fitted carpet**	-7.19e+06	0.38
**Damp spots bedroom**	-1.55e+06	0.86
**Mould inside home**	3.23e+06	0.42
**Mould odour**	3.97e+06	0.22
**Condensation on window**	1.87e+06	0.67
**Water damage**	-3.11e+06	0.38
**Floor level (ref: Ground floor)**		
**First floor**	6.54e+06	0.36
**Higher than first floor**	5.72e+06	0.41

Supplementary table 4: Quantile regression model estimates for bacterial load of indoor airborne microbiome expressed as 16S rRNA/m2. The independent variables that showed p value ≤ 0.25 were kept in the successive regression model.

Variable	16S rRNA/m2	
	B	p value
**Key factors**		
**City (ref: Aarhus)**		
**Bergen**	-1.87e+07	0.09
**Reykjavik**	-1.29e+07	0.03
**Tartu**	1.76e+07	0.14
**Uppsala**	-1.50e+07	0.06
**Season (winter vs summer)**	3.60e+06	0.33
**Occupant factors**		
**Dog (No vs Yes)**	6.13e+06	0.32
**Cat (No vs Yes)**	7.89e+06	0.14
**No of occupants (1 vs ≥ 2)**	1.45e+07	0.001
**Occupant's age**	-3.88e+05	0.13
**Cleaning frequency (ref: < 1 day per week)**		
**1-3 days per week**	5.04e+06	0.14
**4-7 days per week**	1.36e+07	0.02
**Kitchen fan use (ref: Never)**		
**Sometimes**	-4.46e+06	0.65
**All of the time**	-5.35e+06	0.46
**Indoor factors**		
**Open cola heating (No vs Yes)**	-6.45e+06	0.19
**Fitted carpet**	-8.94e+06	0.19

Model 1: key factors. Model 2: key factors and occupant factors. Model 3: key factors, occupant factors and indoor factors.

Supplementary Table 5: Univariate linear model estimates for alpha-diversity of indoor airborne microbiome expressed as richness and Shannon index. All independent variables showed p value ≤ 0.25 in univariate linear regression were used in multivariate linear regression analysis.

Variable	Richness		Shannon	
	**B**	**p value**	**B**	**p value**
**City (ref: Aarhus)**				
**Bergen**	-45.2	0.001	-0.16	0.01
**Reykjavik**	-6.4	0.58	0.03	0.59
**Tartu**	41.3	0.012	0.21	0.01
**Uppsala**	6.2	0.65	0.13	0.07
**Season winter vs summer**	-3.7	0.64	-0.04	0.28
**Dog (No vs yes)**	103.3	0.001	0.38	0.001
**Cat (No vs yes)**	22.2	0.08	0.12	0.06
**No of occupants (1 vs ≥ 2)**	25.4	0.03	0.15	0.02
**Occupant's age**	0.84	0.14	0.007	0.01
**Cleaning frequency (ref: < 1 day per week)**				
**1-3 days per week**	-9.4	0.31	0.003	0.9
**4-7 days per week**	22	0.12	0.15	0.03
**Use of bleach (No vs yes)**	-14	0.13	-0.07	0.12
**Use of ammonia (No vs yes)**	-18.5	0.12	-0.11	0.06
**Window open at night (ref: Never)**				
**Sometimes**	-12.1	0.27	-0.02	0.65
**All of the time**	-23.3	0.01	-0.1	0.03
**Kitchen fan use (ref: Never)**				
**Sometimes**	-25	0.04	-0.13	0.05
**All of the time**	-36.5	0.002	-0.17	0.005
**House age**	0.31	0.02	0.001	0.05
**Mattress age**	0.02	0.9	-0.001	0.84
**Central heating**	22.1	0.01	0.11	0.01
**Electric heating**	-23.7	0.005	-0.11	0.01
**Ducted heating**	-2.4	0.88	0.04	0.64
**Open coal heating**	-22.3	0.07	-0.08	0.19
**Radiators heating bedroom**	30.5	0.001	0.16	0.001
**AC**	-8.1	0.6	-0.07	0.38
**Airbrick bedroom**	15.5	0.21	0.12	0.067
**Fitted carpet**	19.9	0.21	0.015	0.85
**Rug bedroom**	28.4	0.001	0.08	0.07
**Damp spots bedroom**	-0.12	0.9	-0.04	0.72
**Mould inside home**	9.3	0.4	0.06	0.25
**Mould odour**	12.2	0.44	-0.002	0.97
**Condensation on window**	-3.32	0.73	-0.12	0.01
**Water damage**	-3.7	0.67	-0.03	0.41
**No. of rooms (ref: One)**				
**Two**	-2	0.96	0.22	0.29
**Three or more**	-10.6	0.77	0.11	0.56

Supplementary Table 5: continues.

Variable	Richness		Shannon	
	B	p value	B	p value
**Floor level (ref: Ground floor)**				
**First floor**	20	0.38	0.06	0.62
**Higher than first floor**	8	0.72	0.08	0.48
**Bedroom size**	3.07	0.001	0.001	0.03
**Floor heating**	20.3	0.18	0.02	0.76
**Bedroom wallpaper**	3.84	0.71	-0.009	0.86
**Painted fiberglass**	-22.6	0.06	-0.12	0.05
**Wall vent**	-13.5	0.12	-0.04	0.36
**Ceiling exhaust**	-8.03	0.69	-0.02	0.83
**House type (ref: farmhouse)**				
**Detached house**	-49.9	0.07	-0.26	0.08
**Terraced house**	-7	0.87	-0.21	0.42
**Apartment building**	-72	0.01	-0.25	0.09

Supplementary Table 6: multivariate linear regression model estimates for alpha-diversity of indoor airborne microbiome expressed as richness, Shannon index include all independent variables showed p value ≤ 0.25 in univariate linear regression.

Variable	Richness		Shannon	
	B	p value	B	p value
**City (ref: Aarhus)**				
**Bergen**	-54.5	0.01	-0.21	0.12
**Reykjavik**	8.1	0.58	0.06	0.34
**Tartu**	50	0.01	0.29	0.01
**Uppsala**	16.1	0.3	0.1	0.26
**Season winter vs summer**	-10.3	0.21	-0.09	0.04
**Dog (No vs yes)**	109.7	0.001	0.42	0.001
**Cat (No vs yes)**	10	0.41	0.07	0.31
**No of occupants (1 vs ≥ 2)**	29.7	0.01	0.16	0.01
**Occupant's age**	1.4	0.01	0.01	0.003
**Cleaning frequency (ref: < 1 day per week)**				
**1-3 days per week**	-14.0	0.12	-0.01	0.95
**4-7 days per week**	17.9	0.19	0.13	0.05
**Use of bleach (No vs yes)**	11.4	0.24	0.06	0.25
**Use of ammonia (No vs yes)**	10.4	0.43	0.02	0.68
**Window open at night (ref: Never)**				
**Sometimes**	-9.1	0.42	-0.02	0.68
**All of the time**	-14.2	0.15	-0.08	0.13
**Kitchen fan use (ref: Never)**				
**Sometimes**	-10.8	0.39	-0.05	0.40
**All of the time**	-14.6	0.28	-0.06	0.41
**House age**	0.28	0.05	0.002	0.03
**Central heating**	-27.9	0.10	-0.12	0.18
**Electric heating**	6.14	0.71	0.07	0.40
**Open coal heating**	-2.8	0.83	-0.02	0.72
**Radiators heating bedroom**	6.1	0.87	0.08	0.35
**AC**	-3.0	0.83	-0.07	0.38
**Airbrick bedroom**	-1.9	0.95	0.05	0.52
**Fitted carpet**	7.8	0.63	-0.001	0.93
**Rug bedroom**	17.7	0.06	0.004	0.90
**Mould inside home**	2.3	0.85	0.06	0.26
**Condensation on window**	-13	0.18	-0.18	0.001
**Use of bleach (No vs yes)**	11.4	0.24	0.06	0.25

Supplementary Table 7: multivariate linear regression model estimates for alpha-diversity of indoor airborne microbiome expressed as richness and Shannon index using three succussive models. The independent variables that showed p value ≤ 0.25 were kept in the successive regression model. Total number of samples from the five study centres were included in the regression model.

Variable	Richness		Shannon	
	**B**	**p value**	**B**	**p value**
**Key factors**				
**City (ref: Aarhus)**				
**Bergen**	-46.8	0.04	-0.21	0.09
**Reykjavik**	6.3	0.66	0.06	0.44
**Tartu**	54.9	0.01	0.29	0.01
**Uppsala**	21.3	0.23	0.10	0.26
**Season (winter vs summer)**	-10.2	0.23	-0.09	0.04
**Occupant factors**				
**Dog**	108.9	0.001	0.42	0.001
**Cat**	9.9	0.42	0.08	0.23
**No of occupants (1 vs ≥ 2)**	28.5	0.03	0.18	0.01
**Occupant's age**	1.3	0.02	0.009	0.002
**Cleaning frequency (ref: < 1 day per week)**				
**1-3 days per week**	-13.8	0.13	-0.001	0.98
**4-7 days per week**	17.8	0.20	0.15	0.03
**Kitchen fan use (ref: Never)**				
**Sometimes**	-11.0	0.39	-0.07	0.31
**All of the time**	-14.3	0.28	-0.07	0.33
**Indoor factors**				
**House age**	0.3	0.03	0.001	0.03
**Central heating**	-29.7	0.09	-0.12	0.19
**Rug bedroom**	18.9	0.05	0.001	0.97
**Mould inside home**	5.9	0.62	0.12	0.05
**Mould odour**	-4.5	0.78	-0.12	0.11
**Condensation on window**	-8.8	0.37	-0.15	0.004
**Water damage**	-11.7	0.18	-0.07	0.11

Model 1: key factors. Model 2: key factors and occupant factors. Model 3: key factors, occupant factors and indoor factors.

Supplementary Table 8: multivariate linear regression model estimates for alpha-diversity of indoor airborne microbiome expressed as richness and Shannon index. For samples collected in Tartu no EDC questionnaire was available, therefore this regression model included only samples from the other cities. A subset of the samples, (excluding the samples from Tartu) were used in the regression model.

Variable	Richness		Shannon	
	B	p value	B	p value
**City (ref: Aarhus)**				
**Bergen**	-48.3	0.13	-0.15	0.35
**Reykjavik**	34.8	0.05	0.22	0.02
**Uppsala**	22.8	0.23	0.13	0.19
**Season winter vs summer**	-3.6	0.73	-0.05	0.3
**Dog (No vs yes)**	109.1	0.001	0.45	0.001
**Cat (No vs yes)**	-8.1	0.61	-0.002	0.97
**No of occupants (1 vs ≥ 2)**	21.7	0.17	0.08	0.37
**Occupant's age**	1.22	0.09	0.01	0.009
**Cleaning frequency (ref: < 1 day per week)**				
**1-3 days per week**	-18.6	0.09	-0.10	0.11
**4-7 days per week**	11.5	0.49	0.07	0.41
**Use of bleach (No vs yes)**	3.7	0.73	0.01	0.83
**Use of ammonia (No vs yes)**	3.4	0.82	0.06	0.25
**Window open at night (ref: Never)**				
**Sometimes**	-5.8	0.68	0.01	0.71
**All of the time**	-13.1	0.25	-0.05	0.37
**Kitchen fan use (ref: Never)**				
**Sometimes**	0.9	0.95	0.01	0.86
**All of the time**	9.4	0.60	0.03	0.70
**House age**	0.28	0.11	0.001	0.16
**Central heating**	-40.7	0.07	-0.21	0.08
**Electric heating**	0.19	0.99	0.04	0.72
**Open coal heating**	4.7	0.77	-0.01	0.82
**Radiators heating bedroom**	10.0	0.68	0.15	0.24
**AC**	-0.46	0.98	-0.09	0.39
**Airbrick bedroom**	-7.3	0.65	0.04	0.61
**Fitted carpet**	1.24	0.94	0.05	0.61
**Rug bedroom**	7.13	0.55	-0.04	0.46
**Mould inside home**	15.5	0.25	0.08	0.24
**Condensation on window**	-10.4	0.39	-0.13	0.03
**Bedroom size**	2.3	0.001	0.01	0.04
**Floor heating**	24.6	0.21	0.08	0.45
**Painted fiberglass**	-11.8	0.41	-0.07	0.33
**Wall vent**	-28.1	0.01	-0.13	0.04
**House type (ref: farmhouse)**				
**Detached house**	4.3	0.97	-0.03	0.78
**Terraced house**	-4.3	0.82	-0.06	0.65
**Apartment building**	-2.7	0.8	-0.006	0.91

Supplementary table 9: Associations between environmental determinant and microbial communities based on ANOSIM test for categorical variables and Mantel test for continuous variables. R value is scaled between -1 and +1. Zero mean very similar bacterial communities, where 1 means very different bacterial community. Values less than zero indicate that there is more dissimilarity among replicate units within samples than between samples.

ANOSIM Test	R	p value
**City**		
**Aarhus vs Bergen**	0.15	0.001
**Aarhus vs Reykjavik**	0.07	0.002
**Aarhus vs Tartu**	0.15	0.001
**Aarhus vs Uppsala**	0.07	0.001
**Bergen vs Reykjavik**	0.042	0.001
**Bergen vs Tartu**	0.304	0.001
**Bergen vs Uppsala**	0.11	0.001
**Reykjavik vs Tartu**	0.203	0.001
**Reykjavik vs Uppsala**	0.048	0.028
**Tartu vs Uppsala**	0.148	0.001
**Season winter vs summer**	0.009	0.124
**Dog (No vs yes)**	0.29	0.001
**Cat (No vs yes)**	0.057	0.09
**No of occupants (1 vs ≥ 2)**	-0.036	0.84
**Cleaning frequency**	0.032	0.04
**Use of bleach**	-0.02	0.89
**Use of ammonia**	-0.07	0.98
**Window open at night (No vs yes)**	0.02	0.004
**Kitchen fan use**	0.012	0.121
**Central heating**	0.032	0.91
**Electric heating**	0.027	0.92
**Ducted heating**	0.069	0.91
**Open coal heating**	0.071	0.97
**Radiators heating bedroom**	0.0402	0.94
**AC**	0.051	0.85
**Airbrick bedroom**	0.033	0.15
**Fitted carpet**	0.0372	0.203
**Rug bedroom**	0.070	0.002
**Damp spots bedroom**	0.0518	0.741
**Mould inside home**	0.031	0.158
**Mould odour**	0.014	0.375
**Condensation on window**	0.042	0.96
**Water damage**	-0.018	0.83
**Number of rooms (1 vs 2)**	0.02	0.41
**Number of rooms (1 vs ≥ 3)**	0.15	0.04
**Number of rooms (2 vs ≥ 3)**	0.01	0.02
**Wall vent**	0.01	0.002

Supplementary table 10: Associations between environmental determinant and microbial communities-based Mantel test for continuous variables. R value is scaled between -1 and +1. Zero mean very similar bacterial communities, where 1 means very different bacterial community.

Mantel test	R	P value
**House age**	0.045	0.01
**Mattress age**	0.013	0.241
**Occupant's age**	0.043	0.002

Supplementary table 11: Univariate quintile model estimates for endotoxin load (Endotoxin unit/m^2) of indoor airborne microbiome All independent variables showed p value ≤ 0.25 in univariate quintile regression were used in multiple quintile regression analysis.

Variable	Endotoxin unit/m^2	
	B	p value
City (ref: Aarhus)		
Bergen	-933	0.001
Reykjavik	-885.1	0.001
Tartu	933	0.14
Uppsala	-717	0.001
Season winter vs summer	95.6	0.40
Dog (No vs yes)	311	0.11
Cat (No vs yes)	440	0.04
No of occupants (1 vs ≥ 2)	-23.9	0.90
Occupant's age	-11.9	0.14
Cleaning frequency (ref: < 1 day per week)		
1-3 days per week	23.9	0.85
4-7 days per week	119	0.54
Use of bleach (No vs Yes)	-239.2	0.04
Use of ammonia (No vs Yes)	-47.8	0.77
Window open at night (ref: Never)		
Sometimes	23.9	0.85
All of the time	119.6	0.54
Kitchen fan use (ref: Never)		
Sometimes	-287.08	0.13
All of the time	-334.9	0.075
House age	7.31	0.003
Mattress age	5.9	0.53
Central heating	47.84	0.69
Electric heating	122.7	0.75
Ducted heating	-167.46	0.53
Open coal heating	-334.92	0.03
Radiators heating bedroom	143.5	0.22
AC	71.7	0.77
Airbrick bedroom	119.6	0.51
Fitted carpet	167.46	0.46
Rug bedroom	167.46	0.20
Damp spots bedroom	-235.2	0.82
Mould inside home	239.2	0.16
Mould odour	284.6	0.28
Condensation on window	311.0	0.04
Water damage	47.84	0.70
No. of rooms (ref: One)		
Two	382.77	0.58
Three or more	-167.46	0.79

Supplementary table 11: continue.

Variable	Endotoxin unit/m² B	p value
**Floor level (ref: Ground floor)**		
**First floor**	502.3	0.05
**Higher than first floor**	358.8	0.17
**Bedroom size**	9.02	0.33
**Floor heating**	327.7	0.18
**Bedroom wallpaper**	-167.46	0.23
**Painted fiberglass**	95.6	0.57
**Wall vent**	-263.1	0.01
**Ceiling exhaust**	-239.23	0.35
**House type (ref: farmhouse)**		
**Detached house**	-454.5	0.38
**Terraced house**	-741.6	0.77
**Apartment building**	-645.93	0.22

Supplementary table 12: Quantile regression model estimates for endotoxin load of indoor airborne microbiome expressed as Endotoxin unit/m^2 include all independent variables showed p value ≤ 0.25 in univariable quantile regression.

Variable	Endotoxin unit/m^2	
	**B**	**P value**
**City (ref: Aarhus)**		
**Bergen**	-589.3	0.07
**Reykjavik**	-742.3	0.001
**Tartu**	786.2	0.18
**Uppsala**	-633	0.01
**Season winter vs summer**	44.8	0.71
**Dog (No vs yes)**	311.4	0.11
**Cat (No vs yes)**	117.3	0.52
**Occupant's age**	-13.1	0.11
**Use of bleach (No vs yes)**	-62.7	0.57
**Window open at night (ref: Never)**		
**Sometimes**	-69.1	0.83
**All of the time**	-84.3	0.66
**Kitchen fan use (ref: Never)**		
**Sometimes**	-119.4	0.49
**All of the time**	-226.4	0.22
**House age**	5.4	0.03
**Open coal heating**	149.5	0.28
**Radiators heating bedroom**	154.5	0.58
**Rug bedroom**	30.3	0.83
**Mould inside home**	21.4	0.83
**Condensation on window**	121.5	0.90
**Floor level (ref: Ground floor)**		
**First floor**	238.2	0.32
**Higher than first floor**	106.5	0.65

Supplementary table 13: Quantile linear regression model estimates for endotoxin load of indoor airborne microbiome expressed as Endotoxin unit/m2. The independent variables that showed p value ≤ 0.25 were kept in the successive regression model. Total number of samples from the five study centres were included in the regression model.

Variable	Endotoxin unit/m^2	
	B	p value
**Key factors**		
**City (ref: Aarhus)**		
**Bergen**	-916.2	0.05
**Reykjavik**	-856.7	0.001
**Tartu**	710.9	0.26
**Uppsala**	-724.8	0.03
**Season (winter vs summer)**	42	0.73
**Occupant factors**		
**Dog**	268.7	0.18
**Occupant's age**	-14.49	0.10
**Cleaning frequency (ref: < 1 day per week)**		
**1-3 days per week**	-9.85	0.93
**4-7 days per week**	125.2	0.53
**Kitchen fan use (ref: Never)**		
**Sometimes**	-67.3	0.72
**All of the time**	-230.6	0.25
**Indoor factors**		
**House age**	5.5	0.04
**Open coal heating**	-315	0.10
**Floor level (ref: Ground floor)**		
**First floor**	362.42	0.16
**Higher than first floor**	225.6	0.38

Model 1: key factors. Model 2: key factors and occupant factors. Model 3: key factors, occupant factors and indoor factors.

Supplementary table 14: Quantile linear regression model estimates for endotoxin load of indoor airborne microbiome expressed as Endotoxin unit/m^2 include all independent variables showed p value ≤ 0.25 in univariable quantile regression. For samples collected in Tartu no EDC questionnaire was available, therefore this regression model included only samples from the other cities. A subset of the samples, (excluding the samples from Tartu) were used in the regression model.

Variable	Endotoxin unit/m^2	
	**B**	**p value**
**City (ref: Aarhus)**		
**Bergen**	-514.5	0.26
**Reykjavik**	-666.7	0.008
**Uppsala**	-562.5	0.05
**Season winter vs summer**	86.0	0.58
**Dog (No vs yes)**	532.9	0.05
**Cat (No vs yes)**	35.5	0.88
**Use of bleach (No vs yes)**	-147.6	0.28
**Occupant's age**	-18.2	0.07
**Window open at night (ref: Never)**		
**Sometimes**	-110.1	0.58
**All of the time**	-71.9	0.67
**Kitchen fan use (ref: Never)**		
**Sometimes**	-3.87	0.98
**All of the time**	-164.3	0.48
**House age**	8.8	0.01
**Open coal heating**	-298.9	0.12
**Radiators heating bedroom**	143.1	0.74
**Rug bedroom**	65	0.71
**Mould inside home**	-51.1	0.77
**Condensation on window**	-73.6	0.66
**Floor level (ref: Ground floor)**		
**First floor**	368.5	0.23
**Higher than first floor**	238.6	0.44
**Bedroom size**	-3.6	0.76
**Floor heating**	183.0	0.47
**House type (ref: farmhouse)**		
**Detached house**	-1169.2	0.39
**Terraced house**	-1414.4	0.30
**Apartment building**	-1191.0	0.38

Supplementary methods

1.Dust, Endotoxin and DNA extraction

The dust was extracted from EDC clothes as following, an EDC cloth was removed from the plastic envelope, placed in sterile stomacher bag, and 20 ml extraction buffer consisting of pyrogen free water (PFW) (Milli-Q® A10 Ultrapure Water) and 0.05% Tween 20 was added. The samples were then processed in Smasher™ (bioMérieux, Marcy-l'Étoile, France) for 3 min at the fast mode (620 strokes min-1). The extraction fluid was transferred to 50 ml Falcon tube and kept on ice. This procedure was repeated with 30 ml PFW. The total volume of the extraction fluid was ~45 ml. Around 4.5 ml were transferred into a 15 ml Falcon tube, dust particles and cells were removed by centrifugation at 1000 × g for 15 min, and the supernatants were stored in 500 µl aliquots at – 20 oC until LAL assay analysis. The remaining 90 % of the dust-suspension was filtered onto 25 mm 0.22 µm pore size polyethersulfone membrane filters (Merck, New Jersey, United States) using glass filtration equipment that was rinsed with hydrochloric acid and ethanol and autoclaved in between runs. The membrane filters were transferred aseptically into pre-filled bead beating tubes and stored at -20°C until DNA extraction. Unexposed EDC cloths were used as negative controls following the same extraction procedure. The DNeasy PowerSoil Pro Kit (MO BIO Laboratories, a Qiagen Company, Germany) was used to extract DNA from the membrane filters following the manufacturer's instructions with a minor modification, which included prolonged bead-beating using a TissueLyser bead-beating device for 10 min at 50 Hz. All the extractions and were performed between January and October 2020, by the same person at the same facility (Section for Microbiology, Department of Biology, Aarhus University, Denmark), using the same protocol to avoid batch to batch variations.

2.16S rRNA Amplicon Sequencing

16S rRNA genes from the samples were amplified using the bacteria-specific primers, Bac341F (5'-CCT ACG GGN GGC WGC AG-3') and Bac805R (5'-GAC TAC GGT ATC TAA TCC-3') (1). These primers amplify the V3 and V4 regions of the 16S rRNA gene. The Illumina protocol (16S Metagenomic Sequencing Library Preparation) was used for amplification of the 16S rRNA gene. The protocol included three PCR steps. In the first PCR step, the bacteria-specific primers were used to amplify the V3 and V4 regions of the 16S rRNA gene.

The PCR mixture contained 4 µL template DNA, 0.5 µl forward primer (0.2 µM) , 0.5 µl reverse primer (0.2 µM) , 12.5 µl 2 × KAPA HiFi Hotstart polymerase (KAPA Biosystems, Wilmington, MA, United States), 0.5 µl BSA (bovine serum albumin ;4 g/L) and dH2O up to 25 µl. The thermal cycling was as follows: an initial denaturation at 95°C for 3 min, 25 cycles with denaturation at 95°C for 30 s, annealing at 55°C for 30 s, elongation at 72°C for 30 s, and a final elongation step at 72°C for 5 min. In the second PCR step, the Illumina overhang adaptors were added using the same conditions as for the first step, but without added BSA and using only 10 amplification cycles. In the third PCR step, Nextera XT Index primers from the Nextera XT Index kit were used. Each reaction contained 2.5 µL template DNA, 12.5 µl KAPA HiFi HotStart ReadyMix, 5 µl dH2O, 2.5 µl Index primer 1 (N7XX) and 2.5 µl Index primer 2 (S5XX). The same PCR thermal cycling program as for first and the second step was used but with only 8 cycles. AMPure XP magnetic beads were used for cleaning of the PCR products following each PCR step. The Quant-iTTM dsDNA BR assay kit and a FLUOstar Omega fluorometric microplate reader (BMG LABTECH, Ortenberg, Germany) were used to measure the concentration of the PCR products. The samples were pooled and diluted to contain approximately 3 ng/µl DNA. Before sequencing on a MiSeq sequencer (Illumina, San Diego, CA, United States), the DNA concentrations of the pooled samples were quantified using a Quant-iTTM dsDNA BR assay kit and a Qubit fluorometer (Thermo Fisher Scientific, Waltham, MA, United States).

3.Quantitative PCR

The qPCR reactions targeting 16S rRNA genes were carried out in a 20 µl reaction volume containing 2µl DNA template, 10 µl SYBR Green 1Master-2x, 2 µl BSA (10 mg/ml), 1 µl forward primer Bac908F (5'-AAC TCA AAK GAA TTG ACG GG-3') (10 pmol/ml) and 1 µl reverse primer Bac1075R (5'- CAC GAG CTG ACG ACA RCC-3') (10 pmol/ml) (2). Negative Controls were created by replacing the DNA template with ddH2O. Thermal cycling and fluorescence measurements were carried out using an MX3005p qPCR machine (Agilent, Santa Clara, CA, United States). One cycle of initial denaturation at 95°C for 5 minutes was followed by 45 cycles of 95°C for 30 seconds, 56°C for 30 seconds, 72°C for 20 seconds, and 80°C for 7 seconds. Serial dilutions of a plasmid encoding a full-length 16S rRNA gene linked to Sphingomonadales were prepared fresh for every qPCR thermal cycling run to generate standard curves that allow absolute quantification of 16S rRNA genes.

The detection limit was defined as the average of the assay blanks plus two times the standard deviation of these blanks. The results were reported in16S rRNA copies/m2.

4.Raw data processing

The raw data processing was carried out in R version 4.2.1 (3) as following, the primers were trimmed from the sequences using the cutadapt package version 1.16 (4). DADA2 (Divisive Amplicon Denoising Algorithm 2) version 1.18.0 (5) used for modelling and correcting Illumina-sequenced amplicon errors. The ASVs (Amplicon Sequence Variants) were taxonomically classified at species level using the SILVA database version 138 (6). The sequences were subsampled to 20,000 reads using the shortread package version 1.48.0 (7) to ensure accurate richness comparisons, as DADA2 pipeline tends to exaggerate richness estimates linearly with increasing number of reads. The decontam package version 1.10.0 (8) was used to identify and remove contaminant ASVs in exposed EDC cloths compared to the prevalence in negative controls (unexposed EDC cloths and negative PCR controls) using the “prevalence" method and the “isContaminant” function.

References

1. Klindworth A, Pruesse E, Schweer T, Peplies J, Quast C, Horn M, et al. Evaluation of general 16S ribosomal RNA gene PCR primers for classical and next-generation sequencing-based diversity studies. Nucleic acids research. 2013;41(1):e1-e.
2. Ohkuma M, Kudo T. Phylogenetic analysis of the symbiotic intestinal microflora of the termite Cryptotermes domesticus. FEMS microbiology letters. 1998;164(2):389-95.
3. Team RC. R: A language and environment for statistical computing. 2013.
4. Martin M. Cutadapt removes adapter sequences from high-throughput sequencing reads. EMBnet journal. 2011;17(1):10-2.
5. Callahan BJ, McMurdie PJ, Rosen MJ, Han AW, Johnson AJA, Holmes SP. DADA2: High-resolution sample inference from Illumina amplicon data. Nature methods. 2016;13(7):581-3.
6. Quast C, Pruesse E, Yilmaz P, Gerken J, Schweer T, Yarza P, et al. The SILVA ribosomal RNA gene database project: improved data processing and web-based tools. Nucleic acids research. 2012;41(D1):D590-D6.
7. Morgan M, Anders S, Lawrence M, Aboyoun P, Pages H, Gentleman R. ShortRead: a bioconductor package for input, quality assessment and exploration of high-throughput sequence data. Bioinformatics. 2009;25(19):2607-8.
8. Davis NM, Proctor DM, Holmes SP, Relman DA, Callahan BJ. Simple statistical identification and removal of contaminant sequences in marker-gene and metagenomics data. Microbiome. 2018;6(1):1-14.
